# Capacity and capability of remote sensing to inform invasive plant species management in the Pacific Islands region

**DOI:** 10.1111/cobi.14344

**Published:** 2024-08-21

**Authors:** Carrol M. H. Chan, Christopher J. Owers, Sascha Fuller, Matt W. Hayward, David Moverley, Andrea S. Griffin

**Affiliations:** ^1^ Conservation Science Research Group, School of Environmental and Life Sciences University of Newcastle Callaghan New South Wales Australia; ^2^ Earth Sciences, School of Environmental and Life Sciences University of Newcastle Callaghan New South Wales Australia; ^3^ Island and Ocean Ecosystems Programme Secretariat of the Pacific Regional Environment Programme Apia Samoa

**Keywords:** conservation, environmental management, geospatial technology, invasive species, invasive species management, Pacific Islands, remote sensing, conservación, especie invasora, gestión ambiental, islas del Pacífico, manejo de especies invasoras, tecnología geoespacial, telemetría

## Abstract

The Pacific Islands region is home to several of the world's biodiversity hotspots, yet its unique flora and fauna are under threat because of biological invasions. These invasions are likely to proliferate as human activity increases and large‐scale natural disturbances unfold, exacerbated by climate change. Remote sensing data and techniques provide a feasible method to map and monitor invasive plant species and inform invasive plant species management across the Pacific Islands region. We used case studies taken from literature retrieved from Google Scholar, 3 regional agencies’ digital libraries, and 2 online catalogs on invasive plant species management to examine the uptake and challenges faced in the implementation of remote sensing technology in the Pacific region. We synthesized remote sensing techniques and outlined their potential to detect and map invasive plant species based on species phenology, structural characteristics, and image texture algorithms. The application of remote sensing methods to detect invasive plant species was heavily reliant on species ecology, extent of invasion, and available geospatial and remotely sensed image data. However, current mechanisms that support invasive plant species management, including policy frameworks and geospatial data infrastructure, operated in isolation, leading to duplication of efforts and creating unsustainable solutions for the region. For remote sensing to support invasive plant species management in the region, key stakeholders including conservation managers, researchers, and practitioners; funding agencies; and regional organizations must invest, where possible, in the broader geospatial and environmental sector, integrate, and streamline policies and improve capacity and technology access.

## Abstract (es)

Capacidad y potencial de la telemetría para informar la gestión de especies de plantas invasoras en las islas del Pacífico

**Resumen:**

Las islas del Pacífico albergan varios de los puntos calientes de biodiversidad del planeta; sin embargo, su flora y fauna únicas se encuentran amenazadas por las invasiones biológicas. Es probable que estas invasiones proliferen conforme incrementa la actividad humana y se desarrollan las perturbaciones naturales a gran escala, exacerbadas por el cambio climático. Los datos y las técnicas telemétricas proporcionan un método viable para mapear y monitorear las especies invasoras de plantas y orientar su manejo en la región de las islas del Pacífico. Usamos estudios de caso tomados de la bibliografía de Google Scholar, las bibliotecas digitales de tres agencias regionales y dos catálogos virtuales del manejo de especies invasoras de plantas para analizar la asimilación y retos que enfrenta la implementación de la telemetría en la región del Pacífico. Sintetizamos las técnicas telemétricas y describimos su potencial para detectar y mapear las especies de plantas invasoras con base en la fenología de las especies, características estructurales y algoritmos de textura de imagen. La aplicación de los métodos de telemetría para detectar las especies invasoras de plantas dependió en gran medida de la ecología de la especie, la extensión de la invasión y los datos disponibles de imágenes telemétricas y geoespaciales. Sin embargo, los mecanismos actuales de apoyo para el manejo de especies invasoras de plantas, incluyendo los marcos normativos y la infraestructura para datos geoespaciales, operan de manera aislada, lo que lleva a que se dupliquen los esfuerzos y se creen soluciones insostenibles para la región. Para que la telemetría apoye al manejo de especies invasoras de plantas en la región, los actores clave, incluidos los gestores, investigadores, practicantes, agencias financiadoras y organizaciones regionales, deben invertir, en lo posible, en un sector ambiental y geoespacial más amplio, integrar y simplificar las políticas y mejorar la capacidad y el acceso a la tecnología.

## INTRODUCTION

Invasive species are a significant driver of global change, second only to land degradation (Kearney et al., 2019; Sakai et al., 2001). Climate change threatens to accelerate the spread and effects of invasive species faster than natural ecosystems can adjust or evolve (Skarpaas & Shea, 2007). The effects of invasive species represent a substantial economic burden to countries, and from 1970 to 2017, a mean cost of US$226.8 billion per annum was spent managing them (Diagne et al., 2021). This figure is expected to increase as cost estimates become available for more poorly appraised invasive species (Fantle‐Lepczyk et al., 2022) and the effects of climate change are realized (e.g., Hanley & Roberts, 2019).

Invasive species are non‐native species that have been introduced and have established in a new natural or seminatural ecosystem or habitat and are recognized as a threat to native biological diversity (IUCN, 2000; Tobin, 2018). These species are introduced by way of human activity, either deliberate or accidental (Pyšek et al., 2020), and in this review, we expanded this definition to include native weeds that have become dominant across landscapes and negatively affect human health and cause economic or environmental harm.

Long‐term security and effective management of threatened ecosystems at various scales relies on thorough knowledge of local ecosystems and their biodiversity, including the history and distribution of invasive species and understanding of ecosystem response to urban development and climate change and effective management strategies (Newbold et al., 2015). Management actions are therefore improved through access to geospatial and remotely sensed information, such as historical spatial assessments of species distribution and richness (Tittensor et al., 2014; Turner et al., 2003), facilitating rapid detection and risk assessments to support management actions (Reaser et al., 2007). International networks and programs, such as the Global Biodiversity Information Facility (GBIF, 2024), have taken proactive steps to consolidate and catalog species data for strengthened accessibility. This data accessibility, although limited to certain species, encourages the development of decision‐support technology, such as remote sensing, which can be used to identify invasive plant species threats and inform management action (Alvarez & Solis, 2018).

Remote sensing can be used to map and monitor and thereby assist in the management of invasive species (Bolch et al., 2020; Bradley, 2014). Remote sensing facilitates mapping at various spatial scales and can be more cost‐effective compared with traditional field‐based mapping techniques (Niphadkar & Nagendra, 2016). Medium‐ to very‐high‐resolution satellite imagery is used to map weed species detected from above with relatively high classification accuracies (Shendryk et al., 2020). Additionally, Earth observation data sets provide capacity for multitemporal classifications, further supporting management actions by demonstrating changes in invasive species distribution over time (e.g., Arasumani et al., 2021; Shendryk et al., 2020; West et al., 2017). Invasive species mapping requires knowledge of the species’ ecology and biogeography, including environmental factors in the landscape that may determine presence and distribution (Parker et al., 2021), highlighting the importance of building invasive species knowledge. Therefore, mapping invasive plant species is a transdisciplinary approach in which remotely sensed data and analytics are supported by ecological knowledge of invasive species (Bolch et al., 2020; He et al., 2011). For remote sensing technicians, this challenge includes the need to balance cost‐effectiveness of the mapping activity with output accuracy and securing relevant data specific to each invasive plant species for analysis. However, there are many other challenges, which we considered, that eclipse these considerations and impact the capacity to operationalize remote sensing for mapping invasive plant species in the Pacific Islands region.

**FIGURE 1:**
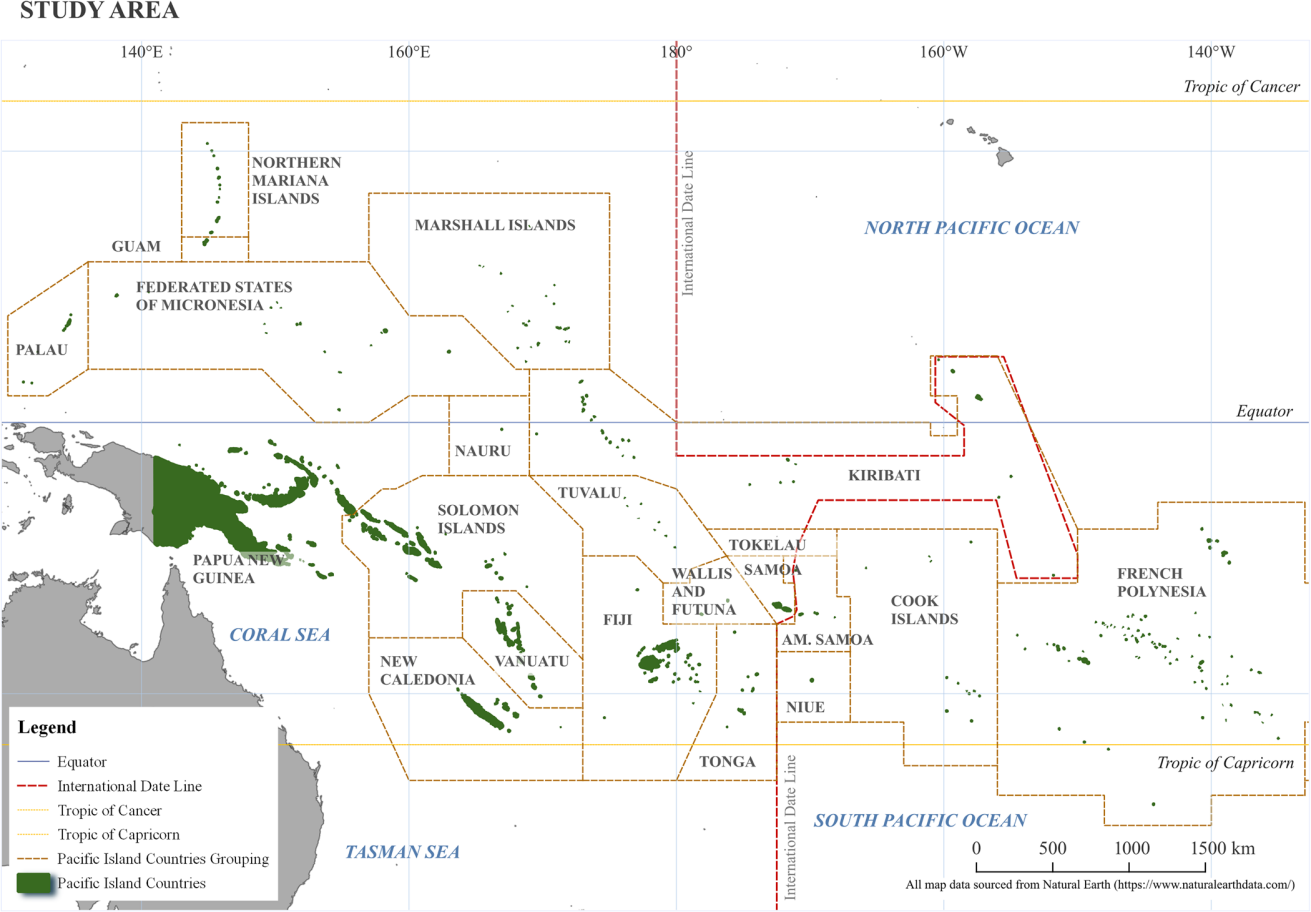
The Pacific Islands region (dark green) and surrounding areas across the Pacific Ocean and the United Nations‐recognized region of Oceania.

The Pacific Islands region encompasses several archipelagoes and vast stretches of ocean that cover one third of Earth's surface (Lenz et al., 2022; Nunn et al., 2016). We define this region as a subregion of Oceania that excludes Australasia (Australia and New Zealand) (Figure 1). This region is a global biodiversity hotspot, and ecosystems have been resilient to natural disturbances and climate change over thousands of years (Keppel et al., 2014). Invasive species are recognized as one of many threats to Pacific resilience (SPREP, 2020a), and this is expected to be exacerbated by large‐scale disturbances. The number of invasive vascular plant species far exceeds the number of native and endemic species in the region, as has been seen in islands, such as Moorea (Meyer et al., 2015; Seebens et al., 2017). From a global context, the Pacific Islands region has had the steepest cumulative increase of naturalized vascular plant species by area (van Kleunen et al., 2015). These introductions are in part due to European colonization, subsequent increased transport and trade (Brock & Daehler, 2021; Seebens et al., 2017), and vulnerable ecosystems that are missing functional groups (Denslow, 2008; Russell et al., 2017). These factors interact with the reality that the successful establishment and spread of invasive plant species results from the dynamic interaction among ecosystem conditions, ecological properties, and population status of the potential invader and anthropogenic disturbance (Meyer et al., 2021).

Management is required to alleviate and mitigate the threats invasive species pose in the Pacific (Keppel et al., 2014). The Secretariat of the Pacific Regional Environment Program (SPREP) *State of Environment and Conservation in the Pacific Islands* report (SPREP, 2020a) identifies growing efforts targeted at the availability and quality of data for baseline and monitoring. However, prioritization of sites for invasive species management in the Pacific Islands region remains a challenge due to the vast ocean space and the financial and human resource limitations of environmental agencies (SPREP, 2020a). Remote sensing of invasive plant species provides numerous advantages to a region where site access and logistics remain challenging. Remote sensing data can be used in large‐scale quantitative analyses to assist adaptive management (Bolch et al., 2020), including site prioritization, detection of emerging effects, and identification of key monitoring targets, which are linked to national strategies and policies, such as the National Biodiversity Strategy and Action Plans (NBSAP) (SPREP, 2009). We sought to critically assess the challenges to and opportunities for the uptake of remote sensing to inform invasive species management across the Pacific Islands region. First, we compiled an overview of invasive plant species management in the Pacific Islands region. Second, we reviewed available regional applications of remote sensing for invasive plant species management. Third, we critically examined existing capacity, including data management, information and communication technology (ICT) and infrastructure, and human resources and policy frameworks, to identify region‐specific factors that limit remote sensing for invasive plant species management. Fourth, we identified remote sensing methods used to map invasive plant species globally. Finally, we devised recommendations to strengthen invasive plant species management capacity that would support remote sensing in the Pacific region. We argue that if provided with much needed institutional support, financing, and technical capacity, Pacific Island nations can use remote sensing to prioritize, design, implement, and monitor invasive plant species to address biodiversity decline and ecosystem collapse.

## LITERATURE SEARCH

We used Google Scholar, 3 regional virtual libraries (SPREP, Pacific Community [SPC], and the University of the South Pacific [USP]), and the Landcare Research and Pacific Geographic Information Systems (GIS)/RS Newsletter catalog. Keywords were used in various combinations to identify relevant literature on invasive plant species (*invasive plant species*, *invasive alien species*, *invasive species*) and remote sensing (*remote sensing*, *satellite imagery*, *Earth observation*, *geospatial*, *GIS*, *mapping*) techniques (*machine learning*, *classification*, *structure*, *texture*) relevant to invasive species management (*management*, *mitigation*, *conservation*) globally and across the Pacific Islands region (*Pacific*, *South Pacific*, *Oceania*). We searched for research articles, reports, and reviews on the topic of using remote sensing to map invasive plant species published from 2016 to 2022. The Landcare Research and Pacific GIS/RS Newsletter catalogs were searched manually, and additional literature and information were searched using cross‐references of selected articles and reports. A total of 3651 results were obtained. We assessed their appropriateness based on title, keywords, and abstract contents. The papers that seemed most relevant to our aims (477 papers) were then read in full. We removed papers that were not relevant, which left 116 papers in total. Among these, those focusing on invasive plant species in the Pacific (42 papers) are listed in Appendix S1.

## INVASIVE SPECIES AND THEIR MANAGEMENT IN THE PACIFIC REGION

Invasive species are the second most common threat associated with species extinction worldwide (Bellard et al., 2016). From 2 invasive species assessments that have taken place in the Pacific region, the current number of invasive species is expected to exceed the number of native and endemic species. These findings are in respect to land area (van Kleunen et al., 2015) and in comparison to native flora (Meyer, 2014). Of these introductions across the region, relatively few have been studied or recognized as a species of concern. At least 2834 known invasive plant species across the Pacific Islands region are listed in the Pacific Islands Pest List Database (SPC, 2023). To date, management actions are ongoing and focused on species that pose economic, health, and environmental harm.

Invasive species that affect human livelihoods and health negatively warrant greater concern and urgent management intervention (Dovey et al., 2004). Of at least 500 plant species introductions in Samoa, high‐risk plant invasive species include African tulip (*Spathodea campanulata*), merremia vine (*Decalobanthus peltatus*), African rubber tree (*Funtumia elastica*), and mint weed (*Mesosphaerum pectinatum*) (Government of Samoa, 2019). Control and eradication programs have been undertaken for these species with varying results at site or national level. For example, merremia management has focused on site containment as biocontrol agents are being investigated for Pacific Island countries where the vine is a management priority (Paynter et al., 2006). This site containment management approach is similar for taro vine (*Epipremnum pinnatum*) on Niue, where biocontrol agents are also being investigated (McGrannachan et al., 2021). Other priority invasive plant species that have largely been contained on Niue include Singapore daisy (*Wedelia trilobata*) and Honolulu rose (*Clerodendrum chinense*) (Government of Niue, 2012).

Despite the threat of invasive species to Pacific biodiversity (SPREP, 2020a), the peer‐reviewed literature on the region from which policy makers and practitioners can draw is limited (Appendix S1). Instead, Pacific Island nations rely on observations and expertise from local and regional researchers, practitioners, community leaders, regional technical advice, workshops, personal communication, and reports to prioritize invasive species and management actions (Day & Winston, 2016). Prioritization made at the local level can entail a bias favoring livelihood security over the protection and conservation of native biodiversity (Brodie et al., 2013; Lenz et al., 2022). Ensuring risk assessments are comprehensive and unbiased would support more balanced management actions that safeguard both livelihoods and native biodiversity.

The SPREP is a key regional player in providing technical advice and support for national practitioners and policy makers for the management of invasive species (SPREP, 2000). Mandated by Pacific Island governments to promote cooperation and provide technical assistance to strengthen environmental conservation and management, SPREP supports regional partnerships, including the Pacific Invasive Partnership (PIP), Pacific Invasives Learning Network (PILN), and Pacific Regional Invasive Species Management Support Services (PRISMSS). These partnerships facilitate access to evidence‐based science, assist with developing national strategies, awareness programs, eradication and restoration projects, and prioritize data accessibility. The endorsement of the *Guidelines for Invasive Species Management in the Pacific* by the member countries of SPREP and SPC in 2009 (SPREP, 2009) ratified regional recognition of the problem. Since then, 9 countries have developed national invasive species strategy and action plans (NISSAP) (SPREP, 2020a). Several regional frameworks and policies focused on biodiversity conservation have also been developed collaboratively and endorsed (Appendix S2) (Jupiter, Mangubhai, et al., 2014). Such initiatives have furthered peer‐to‐peer learning; coordination of successful biocontrol programs, such as broomweed (*Sida acuta*) and mile‐a‐minute (*Mikania micrantha*) in Fiji, Papua New Guinea, and Vanuatu (Day & Winston, 2016; SPREP, 2020b); and the development of regional resources that include the Pacific Islands Pest List Database and the Pacific Biodiversity Information Facility (PBIF) (Lenz et al., 2022; SPREP, 2016, 2020a). Although these instruments and initiatives reflect the region's willingness to engage actively with invasive species management, alone they do not provide effective management.

The status of invasive species management in the Pacific region has been reported as “poor to fair*”* (SPREP, 2020a). In 2020, 116 priority invasive plant species management programs were reported, of which 8 resulted in eradication, and there were 67 instances of biocontrol being used (SPREP, 2020a). For biocontrol, these efforts have only slightly increased since 2016, when 62 biocontrol agents were introduced to control 21 invasive weeds, including mile‐a‐minute, giant sensitive tree (*Mimosa diplotricha*), and lantana (*Lantana camara*), across 17 Pacific Island countries (Day & Winston, 2016). Biocontrol was introduced to Fiji, Vanuatu, and Papua New Guinea, and several weed species were reported to be under control after intervention, including the giant sensitive tree, broomweed, and arrowleaf sida (*Sida rhombifolia*) (Day & Winston, 2016). Site prioritization, particularly protected areas, for direct multiple invasive species management is still poor (SPREP, 2020a). Although management efforts focus solely on priority species, the risk of invasive species introductions is still high across the region (see Turbelin et al., 2017; van Kleunen et al., 2015) and occurring at underreported rates (see Laginhas et al., 2023). These continuous introductions and expansions are in part driven by socioeconomic activities (Brock & Daehler, 2021), of which tourism is a large commodity in the Pacific (ADB, 2018). The Pacific Islands region is projected to have a 21% relative increase in emerging invasive species across taxonomic groups from 2005 to 2050 (Seebens et al., 2022).

The available body of published research on the Pacific region suggests fragmented invasive species research, management, and governance, particularly around the monitoring and evaluation of management activities. Recently, Lenz et al. (2022) reiterated Meyer's (2014) call to strengthen Pacific governance for invasive prevention and control. They suggest, at the national level, management efforts be combined with planning activities to attract support through legislation, funding, and multisectoral integration. These activities should aim to support invasive species monitoring through increasing awareness, deliver education, build capacity, strengthen governance, and implement effective action and evaluation programs. We argue that remote sensing, with the right support, is well placed to assist with invasive plant species management (i.e., monitoring, action, and evaluation) in the Pacific Islands region.

## APPLICATION OF REMOTE SENSING TO INVASIVE SPECIES MANAGEMENT IN THE PACIFIC

The earliest documented use of GIS for invasive species in the Pacific Islands region was in 1995 (Poidevin, 1995); however, recurring national interest to improve monitoring and mapping efforts through GIS in NBSAP (Government of Niue, 2012; Government of Solomon Islands, 2016) indicates stagnant development. From the available literature of invasive plant species and related management in the Pacific Islands region (Appendix S1), only 7 sources described the use of remotely sensed data and geospatial tools, of which 4 utilized remotely sensed data and 2 applied classification algorithms to detect and map invasive plant species for management. This indicates limited application of remote sensing and broader geospatial technology for the management of invasive species in the Pacific Islands region. We examined the 2 studies by Pouteau et al. (2013) and Asia Air Survey (2014) that used remote sensing to highlight current and common practices used to detect and prioritize invasive species in the region.

Multisource remotely sensed imagery has been applied to detect dominant invasive tree species on the island of Moorea in French Polynesia. Pouteau et al. (2013) investigated landscape fragmentation by classifying multisource remotely sensed data: multispectral imagery (Quickbird‐2 (0.60‐m resolution)), synthetic aperture radar (SAR) data (StripMap TerraSAR‐X (2.75‐m resolution), JPL/AirSAR (5‐m resolution)), and a digital elevation model (DEM), trained with field data. The classification method, support vector machine (SVM) (definition in Appendix S4), discriminated dominant plant classes that were spectrally distinct; however, the authors did not report evaluation metrics. The use of multisource images provided an advantage, overcoming challenges, such as persistent cloud cover, heterogenous terrain, and dense vegetation, that are usually associated with tropical high volcanic islands (Meyer et al., 2015). The output was used to assess landscape fragmentation where higher fragmentation occurred at lower elevations, and upland landscapes were preserved. This result was one part of a 5‐year research program, the Moorea Biocode Project, which aimed to barcode the entire marine and terrestrial biota and included identifying the spatial distribution of native, naturalized, and invasive plant species (Meyer et al., 2015).

The only other publication, to our knowledge, for which remote sensing was used in the Pacific Islands region for invasive species research was undertaken in O Le Pupu‐Pue National Park in Samoa (Asia Air Survey Co Ltd., 2014). A machine learning classification, random forest (Appendix S4), was used to map merremia. Training data, consisting of field data and visually interpreted sample points, were used to classify high‐resolution WorldView‐2 multispectral imagery at a reported accuracy of 90%. Although little spectral confusion was observed between merremia and the 2 other classes (forest and nonforest), no further studies have been undertaken. The results were utilized to develop site‐specific recommendations for ecological restoration based on the density of the invasive weeds. Further work, such as community consultations to design operational plans for the restoration of the national park, have been undertaken (Atherton, 2015) that considered the results of the 2014 study.

Geospatial analysis and comprehensive field surveys are far more commonly used to determine the threat of invasive species in the Pacific Islands region. Takeda (2010), Keppel et al. (2021), and Lowry et al. (2020) investigated the composition of invasive plant species and their distributive effects across Fiji. As an area of conservation significance, the remaining natural areas of the Sigatoka Sand Dunes were degrading at a much faster rate due to vegetation changes caused by natural disturbances (Takeda, 2010). Similar findings were discovered across the periurban outskirts of Suva through a multivariable regression that showed the abundance of invasive plant species is influenced by the urban–rural sector (Lowry et al., 2020), and in Abaca village, where invasive plant species are threatening endemic species, such as *Pterocymbium oceanicum*, the abundance of these species must be monitored to mitigate the development of monospecific and characterless vegetation as endemic flora are lost (Keppel et al., 2021). Other methods include recording field occurrences of invasive plant species with GPS receivers to map the extent of infestations (US Forest Service, 2020). Although the application of remote sensing for the detection and monitoring of invasive species has been limited in the Pacific Islands region, interest in utilizing the technology is evident from mid‐1990s, when the dominance of African tulip and common guava (*Psidium guajava*) was delineated with Landsat satellite imagery in Fiji (Poidevin, 1995).

## CHALLENGES AND OPPORTUNITIES FOR INVASIVE SPECIES MANAGEMENT IN THE PACIFIC

The limited uptake of remote sensing for identifying and mapping invasive plant species suggests existing challenges in the Pacific. Challenges identified to strengthen collaboration between remote sensing researchers and invasive species practitioners to advance the detection and monitoring of invasive species center on the existence of expertise (Parker et al., 2021). The “great divide” between researcher and practitioner collaboration may be a factor, but alone it does not adequately account for the array of challenges facing the Pacific Islands region (Brewington et al., 2023; Jupiter, Mangubhai, et al., 2014; Parker et al., 2021). We considered the status of infrastructure, which includes regional and national policy frameworks, data availability, technology (including ICT infrastructure), and human capacity, that exists to support or challenge the uptake of remote sensing for invasive species management.

### Pacific Islands policy framework for invasive species management

There are several policy frameworks related to biodiversity and invasive species management (Appendix S3). We examined the key international and regional frameworks and national policies that could be further leveraged to support the use of remote sensing to deliver evidence‐based decision‐making for invasive species management.

Adopted in December 2022, the Kunming–Montreal Global Biodiversity Framework builds on the Strategic Plan for Biodiversity 2011–2020 and integrates broad‐based actions in line with United Nations Sustainable Development Goals (CBD, 2022). The framework emphasizes action that is results oriented, responsible, and transparent at the national level and guides implementation actions that would benefit NISSAP and NBSAP. Recognizing the role of science and innovation in implementation of actions, remote sensing is a suitable solution to support achieving target 6 of the Kunming–Montreal Global Biodiversity Framework (CBD, 2022, p.10), that is, identifying, managing, and, where possible, preventing the introduction and establishment of invasive species to safeguard biodiversity and ecosystem services.

The Integrated Geospatial Information Framework provides a basis to create and implement an effective mechanism to maximize geospatial information management and related resources to solve societal and environmental problems at the national level (UN‐GGIM, 2023). Key to the framework is multisectoral and multiministry access and collaboration to bridge the geospatial digital divide and safeguard socioeconomic prosperity (Bäckstrand, 2006).

Adopted in 2020 after extensive consultation across the region, the Framework for Nature Conservation and Protected Areas in the Pacific Islands Region provides guidance on the region's key priorities for biodiversity conservation and ecosystem management (SPREP, 2021). These priorities are linked to the Global Biodiversity Framework and sustainable development goals to allow for successful implementation at the country level. The framework recognizes invasive species as a priority for action, emphasizing key challenges, such as the need for effective knowledge and skill sharing across the region and for best practices that involve local communities in the decision‐making process. The framework reinforces and emphasizes the collaboration required among government, local communities, development partners, and other key stakeholders.

The fragmentation of available information across the region has led to various information silos affecting the quality and effectiveness of evidence‐based decision‐making across industries, including invasive species management. National Geospatial Data policies aim to support and centralize the availability and quality of data to inform and safeguard national socioeconomic resources, including the management of invasive species (Groom et al., 2017). In 2020, Vanuatu was the first country in the region to develop and endorse a National Geospatial Data Policy (SPC, 2021), which sought to improve centralization and coordination activities at the national level (Willmer, 2021). Tonga and Fiji have made significant headway toward incorporating the Integrated Geospatial Information Framework in respective national plans (SPC, 2022).

The NISSAP adopts regional guidelines for invasive species management to address invasive species at the national level. The NISSAP, managed by national government environmental departments, identifies priorities of invasive species issues, seeks and supports cross‐sectoral collaboration and coordination with all stakeholders, and further identifies resources required for implementation. Of the 9 NISSAP in the region (Appendix S3), 3 are current: Cook Islands, Samoa, and Tonga. Across these strategies, there is no explicit focus on the utilization of remote sensing for invasive species management nor does any such initiative exist. However, there is an emphasis on improving the capture and management of geospatial data.

### Implementation of activities

A common theme across international, regional, and national frameworks and policies is the need for multisectoral collaboration and the inclusion of local communities as stakeholders to successfully implement activities. Regional and national efforts in invasive species management are dominated by regional and large international organizations (Keppel et al., 2012). However, the absence of instruments to govern institutional arrangements runs the risk of overlooking resources needed for local cooperation and management (Jupiter, Jenkins, et al., 2014; Worthy & Race, 2023). Instead, resources are dedicated to creating surface‐level institutional arrangements focused on achieving project‐funded objectives, that is, creating short‐term benefits (Britton, 2000; Keppel et al., 2012). A key absence in these biodiversity and conservation frameworks is the prioritization of research to develop monitoring tools, including remote sensing.

### Access to data and technology

The capacity to respond to the threat of invasive species requires timely access to high‐quality data (Groom et al., 2017; Wallace et al., 2016). Yet, access to data, data quality, and data sharing are challenges that persist in the region (Steven et al., 2019). Although there are numerous regional data systems available (Appendix S2), PBIF is the only regional resource that stores biodiversity data, including invasive species data.

In the case of satellite imagery, intermittent commercial high‐resolution multispectral satellite imagery from vendors, such as MAXAR, has been purchased for areas of interest across the Pacific Islands region (Appendix S2). However, the cost to purchase temporal sequences, potentially prohibitive licensing agreements, and ICT infrastructure required to access and manage such large data sets are considerable restrictions for an already resource‐stretched Pacific region (Steven et al., 2019). Where access to consistent cloud‐free satellite imagery limits usable optical imagery, SAR, known to penetrate clouds, has been suggested as an alternative; however, investment and interest in the region are unknown (SPC, 2019; Steven et al., 2019). These challenges have led to investments in remotely piloted aircraft systems (RPAS) in the region (e.g., Bonte‐Grapentin et al., 2017). Today cloud computing platforms, such as Google Earth Engine (GEE), Microsoft Planetary Computer, and Amazon Web Services’ dedicated cloud Earth observation, have been favored to deliver large‐scale remote sensing solutions (Amani et al., 2020). These platforms reduce barriers to access remote sensing infrastructure and are especially valuable for Pacific Island countries where current ICT infrastructure is inadequate. Regional leaders have emphasized the critical need for Pacific ownership of an Earth observation initiative in the region (GEO, 2019). To address this need, Digital Earth Pacific (DEP), a regional Earth observation initiative, seeks to improve access to remotely sensing solutions, including analysis‐ready data, for regional decision‐makers (SPC, 2021). However, investment, governance, and monitoring instruments are disproportionate across the region and can hinder the progress of such Pacific‐led initiatives (Brodie et al., 2013; SPC, 2019).

The technical infrastructure, human capacity, and funding to support these related data systems at the national level are inconsistent. Pacific Island nations face challenges related to coordinating centralization and improvement of current data management and related technical infrastructure. In larger countries, such as Fiji, access to data and the infrastructure for data sharing and distribution are key challenges (Pene, 2006). Because data and technology are fragmented across organizations, effective distribution across public and private organizations is further hindered by internal policies and procedures because centralized frameworks and policies for data sharing are limited or nonexistent. Smaller countries, such as Kiribati and Marshall Islands, face challenges related to ICT infrastructure, including internet connectivity and prohibitive costs to access tools and data sets online (SPC, 2019). Such challenges can inhibit decision‐making at the national level, requiring unique technical skills to deliver solutions with limited resources (Steven et al., 2019).

### Technical capacity to support remote sensing for invasive species management

Capacity development for biodiversity conservation, including information collection, monitoring, and maintenance, has been identified as a priority in NBSAP, such as Niue (Government of Niue, 2015) and Fiji (Government of Fiji, 2020). However, developing remote sensing capacity is not explicitly stated or supported. The low number of publications for which remote sensing was applied to invasive species management in the Pacific Islands region alludes to the limited availability of regional expertise and resources dedicated to this field (Appendix S1). Where project‐funded remote sensing training opportunities are provided, activities have been limited to key stakeholders, often government department representatives. This can restrict capacity to interested and current practitioners in the invasive species field because emphasis is placed on funder priorities rather than the benefit to practitioners and the research community in general (Britton, 2000).

## REMOTE SENSING METHODS APPLIED TO INVASIVE SPECIES GLOBALLY

The application of remote sensing in invasion science has produced a variety of novel approaches that incorporate species phenology (reoccurring seasonal biological events in plants, such as flowering and leaf development), structural characteristics (e.g., vertical structural measurements, including height, foliage, and canopy cover), and textural information (i.e., distinguishable patterns among a group of image pixels that can differentiate between vegetation classes) (Dronova et al., 2017; Huang & Asner, 2009). These techniques have matured to focus on identifying and incorporating plant traits, spatial patterns, and spectral and spatial properties (Niphadkar & Nagendra, 2016).

**FIGURE 2:**
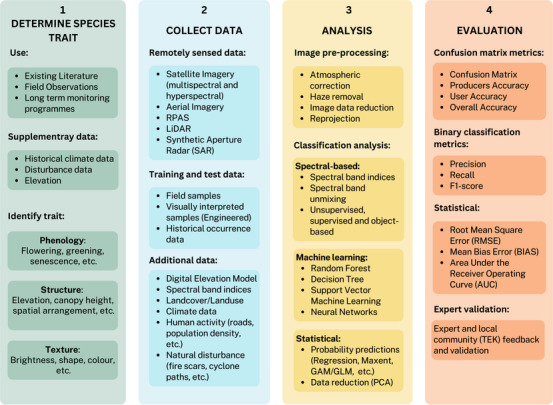
Trade‐offs among the spatial, spectral, and temporal resolution of remotely sensed image data for invasive plant species detection. Figure redrawn and adapted from Howey et al. (2020).

To best utilize available remotely sensed imagery, a strong understanding of the species ecology helps in designing species‐specific remote sensing approaches. Crucial to this design is understanding the trade‐offs that exist among the spatial, spectral, and temporal resolution of remotely sensed data (Bolch et al., 2020). Several types of considerations are required when selecting remotely sensed data for the detection of invasive plant species (Figure 2). The spatial resolution, the smallest feature that can be detected from above, determines the overall mapping scale and extent. Landsat8 offers moderate spatial resolution (30 m) that can detect invasive plant species at the national and regional scale. Although the frequency of image recapture for the same area (temporal resolution) may be high for the Landsat constellation of sensors, plant invasions must be contiguous and widespread for spectral and spatial detection (Bradley, 2014). The spectral resolution of imagery (i.e., width of bands recording information) determines the ability to detect spectral differences and therefore features across the landscape (CRCSI, 2017). These differences are detected using spectral‐based classifications. From these trade‐offs, it can be inferred that high‐spatial‐resolution multispectral imagery (<10 m) is ideal for detecting individual species and early infestations. However, the temporal resolution and spatial coverage of high‐spatial‐resolution data sets are limited. This is because of budgets, technical capacity, infrastructure, and mapping scale constraints.

**FIGURE 3:**
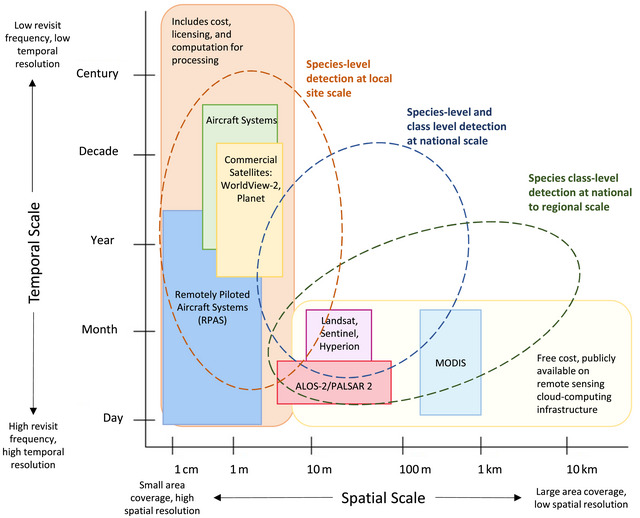
Workflow to map invasive plant species with remote sensing: step 1, determine species trait that can be mapped; step 2, collect data, which requires an examination of existing remotely sensed data; step 3, analysis, in which data processing depends on type of classification method used; step 4, evaluation to determine the accuracy of the mapping.

Here, we examined 3 ecological characteristics that can be seen from above—phenology, texture, and structure—and are used to map invasive plant species (Figure 3). We also examined recent studies relevant to the detection of invasive species applicable to the Pacific Islands region (Table 1). Relevant classification algorithms and spectral indices are described in Appendix S4.

**TABLE 1:** Invasive plant species traits, details, and associated classification methods and remotely sensed data that have been applied successfully to map invasive plant species and relevant studies.

	Traits	Details	Classification methods applied	Commonly used remotely sensed data	Relevant studies
Phenology	Leaf shedding, senescence	Late or early shedding of leaves. Gradual deterioration of plant including die‐off.	Machine learning (RF) Spectral indices (NDVI, NDWI, EVI, VARI) Spectral differences (preflowering, blooming, postflowering) Spectral unmixing Deep learning (CNN) Temporal change detection Species distribution modeling with spectral bands and indices	Multispectral aerial imagery (e.g., RPAS‐mounted multispectral camera) Multispectral satellite imagery (e.g., WorldView‐2, Planet Scope, Landsat constellation, Sentinel‐2, MODIS) Hyperspectral satellite imagery	Boyte, Wylie, & Major, 2015; Bransky et al., 2021; Weisberg et al., 2017
	Plant flowering	Various stages of plant flowering, unique color characteristics, and seasonal blooms can provide distinct pigmentation. Spectral response across plant flowering stages has been detected through spectral indices across temporal sequences of imagery.			Domingo et al., 2023; Lake et al., 2022; Paz‐Kagan et al., 2019; Weisberg et al., 2021
	Seasonal response and change	As seasons change, plant composition differs (producing seeds, new tissue, etc.) from surrounding vegetation; includes leaf coloring, green up, and response to precipitation, droughts, and so forth; to assess seasonal changes, temporal sequences of imagery are required.			Dahal et al., 2022; Elkind et al., 2019; Labonté et al., 2020; West et al., 2017
Texture	Class differentiation	Differentiation among vegetative classes, instead of at a species level; grouped by land cover classes (majority group of species is identified, e.g., cheatgrass infestations); target species class must be spread contiguously over an area that can be detected by the spatial resolution of the remotely sensed imagery; for this trait, medium to high spatial resolution imagery, such as Sentinel‐2 and Landsat, has been applied.	Classification: unsupervised, single‐run supervised, hierarchical OBIA Machine learning (RF, SVM) Dimensionality‐reduction algorithms (PLS‐DA) Edge‐preserving spectral‐smoothing Sequential additive modeling Second‐order texture parameters (GLCM)	Multispectral satellite imagery (e.g., IKONOS, SPOT‐6, Sentinel‐2, Quickbird) Radar imagery SAR (e.g., StripMap TerraSAR‐X, AirSAR) Multispectral aerial imagery (e.g., RPAS‐mounted multispectral camera) Lidar (e.g., aerial laser scanning system mounted camera on aerial system)	Brewer et al., 2022; Laba et al., 2010; Lottering et al., 2020; Pouteau et al., 2013
	Species texture (color, shape, brightness)	Identification of target species based on texture and surrounding vegetation; species texture can be derived using the spatial and spectral information available; masking can also be applied using NDVI or other phenological methods to carry out thresholding.			Baron & Hill, 2020; Chetty et al., 2021; Marzialetti et al., 2021; Mielczarek et al., 2023; Wu et al., 2019
Structure	Canopy structure (overstory, understory)	Leaf shape can affect size of an invasive canopy; canopy height can differentiate invasive trees among surrounding vegetation; LiDAR and airborne laser scanning data are also fused or complimented with the usage of multispectral or hyperspectral data.	Machine learning (RF, SVM) Spectral indices Spectral angle mapper Echo classification Regression models	Multispectral satellite imagery (e.g., WorldView‐2, Landsat constellation, Sentinel‐2) Multispectral aerial imagery (e.g., RPAS‐mounted multispectral camera) Lidar (e.g., aerial laser scanning system mounted camera on aerial system)	Dash et al., 2019; Kattenborn et al., 2019; Lopatin et al., 2019; Marcinkowska‐Ochtyra et al., 2018
	Topographic variables (elevation, slope, aspect)	Elevation, slope, aspect, and hill shade play a role in presence of invasive trees; surrounding vegetation can be masked based on structural characteristics of target species and further detected using these variables and classification techniques.			Chiang & Valdez, 2019; Dai et al., 2020; Paz‐Kagan et al., 2019

Abbreviations: CNN, convolutional neural network; EVI, enhanced vegetation index; GLCM, gray‐level co‐occurrence matrix; Lidar, light detection and ranging; MODIS, moderate‐resolution imaging spectroradiometer; NDVI, normalized difference vegetation index; NDWI, normalized difference water index; OBIA, object‐based image analysis; PLS‐DA, partial least squares‐discriminant analysis; RF, random forest; RPAS, remotely piloted aircraft systems; SAR, synthetic aperture radar; SPOT‐6, satellite pour l'observation de la terre; SVM, support vector machine learning; VARI, visible atmospherically resistant index.

### Phenological‐based detection

Plant phenology refers to specific life cycle events, such as vegetation senescence, green‐up, flowering, and fruit and seed production (Dronova & Taddeo, 2022). These events are identified by spectral changes that occur within the visible and near‐infrared region of the electromagnetic spectrum in optical imagery (Weisberg et al., 2021). Spectral bands that are sensitive to chlorophyll absorption, plant inflorescence, and other plant phenological features are identified through spectral indices (Marcinkowska‐Ochtyra et al., 2018; Zeng et al., 2022). Spectral indices combine 2 or more spectral bands to highlight a measured reflectance or absorption feature, indicating specific features or processes and minimizing background effects (Bolch et al., 2020; Montero et al., 2023) (examples in Appendix S4). Through spectral indices, spectral changes of the invasive plant species and surrounding plant communities’ phenology are detected effectively across temporal sequences of remotely sensed imagery. These traits, when detected, are applied to appropriate classification techniques to map their distribution (Bransky et al., 2021; Elkind et al., 2019; Labonté et al., 2020). These studies are highlighted in Table 1. Cheatgrass (*Bromus tectorum*) is a well‐studied example of plant detection through phenology across time and highlights the importance of repeated data acquisition across seasons (Boyte, Wylie, Major, et al., 2015; Bradley & Mustard, 2005; Bradley et al., 2018), a critical consideration for mapping invasive species in the Pacific region because these are costly to obtain. However, public programs, such as NASA's Landsat program, provide temporally relevant data for detecting widespread invasions and can, instead of high‐resolution data, be optimized through platforms, such as GEE, to reduce computation and related technological infrastructure costs.

### Texture‐based detection

Image texture is another characteristic to exploit in the detection of invasive plant species. Texture features identify spatial patterns and the relationships between neighboring image pixels (Löfstedt et al., 2019). These patterns include distinct color, brightness, or elevation‐derived slope, creating spatial clusters across a landscape (Bradley, 2014; Chetty et al., 2021). Gray‐level co‐occurring matrix (GLCM), or second‐order texture parameters, characterizes image texture by the frequency of occurrence of pixels with specific values in a specific spatial relationship (Löfstedt et al., 2019). The statistical measures extracted from the matrix are used in classification applications (Brewer et al., 2022; Lottering et al., 2019; Tsai & Chou, 2006). Textural features can be integrated with spectral indices to improve overall classification performances. Combined with spectral indices and structural features derived from remotely sensed images, image texture can simplify canopy structure (Brewer et al., 2022) and reduce noise and background disturbances (Xu et al., 2022) and thus produce high classification accuracies in discriminating invasive weeds (Deur et al., 2020; Mielczarek et al., 2023) (Table 1). The SVM classifier has effectively detected target invasive species across landscapes with nonuniform and nonspectrally unique classes (Liang et al., 2020; Sabat‐Tomala et al., 2020). In contrast, random forest classification performs better at differentiating species among homogenous classes (Koerner et al., 2022). Such comparisons help optimize workflows for discriminating invasive species across landscapes in the Pacific, which are small and diverse. A challenge to consider for Pacific implementation is the management of large volumes of data created from texture parameters and the associated computation of optimal feature selection. We therefore suggest that image texture parameters be incorporated where spectral information is not sufficient and to improve classification accuracy.

### Structure‐based detection

Distinctive structural traits, including canopy cover, height, and foliage, help separate a target species from the surrounding landscape (Niphadkar & Nagendra, 2016). Structural traits of invasive plants often dictate their spatial grouping, growing either as independent units or aggregated patches. Elevation and lidar (light detection and ranging) point cloud data have been combined with spectral data to leverage the structural characteristics of invasive grasses and shrubs (Chance et al., 2016; Marcinkowska‐Ochtyra et al., 2018), understory vegetation (Singh et al., 2015), and forest stands (Asner et al., 2008; Dash et al., 2019; Mielczarek et al., 2023). Structural characteristics focused on elevation and canopy height cannot differentiate the target species alone and perform best when combined with spectral and textural information where available. The increased adoption of RPAS in Pacific Island countries, such as American Samoa (Goldfarb, 2019), can be explored to derive structural information, such as elevation (Hauglin & Ørka, 2016) and canopy height (Abeysinghe et al., 2019; Dash et al., 2019), for mapping of invasive plant species (Table 1). Another alternative way to identify vegetation structure is the use of SAR data because optical disturbances, such as cloud cover and shadows, are not captured. Detected information of surface targets, such as shape, moisture, and roughness, is captured by SAR and useful for discriminating vegetation composition (Chen et al., 2010; Ghulam et al., 2014). The application of SAR in the Pacific is currently unknown (Steven et al., 2019). However, the persistence of coverage and terrain challenges that limit RPAS capture and resource constraints associated with acquiring lidar mean there exists an opportunity to exploit SAR as a structural parameter data set for mapping invasive species at larger spatial scales. The studies in Table 1 identify synergistic approaches that use structural information with multiple sensor image data to detect invasive plants.

## REMOTE SENSING OF INVASIVE PLANT SPECIES IN THE PACIFIC ISLANDS

Remote sensing provides the potential to map and monitor plant invasions across landscapes over time. The current global body of literature demonstrates the vast application of data sets and methods used to detect and map invasive species relying on distinct characteristics that include phenology, structure, and texture. These characteristics highlight the opportunity to optimize invasive species management by strengthening collaboration between remote sensing researchers and invasive species practitioners. Where resources to acquire remotely sensed imagery may be limited, public programs (Table 1) that offer historical image archives, such as the Landsat and Sentinel constellation and RPAS, which are widely available across the region, have been successfully utilized. The application of remote sensing to inform invasive plant species management should be fit for purpose (Figure 3), and its usefulness depends on the invasive plant species and its distinct characteristics, the extent of the invasion, and the type of remotely sensed imagery available, particularly the spatial, spectral, and temporal resolution.

There has been steady growth and demand for remote sensing science in the Pacific. Although geospatial developments started in the early 1990s in Fiji (Davis, 1993), concerted efforts to develop remote sensing capacity began with the formalization of the Geospatial Degree Program in 2015 by the region's university, USP. Although global platforms, such as GEE, have become accessible for large‐scale analyses, DEP and other regional initiatives, such as the Pacific Data Hub and PBIF, should be leveraged to strengthen local buy‐in and collaboration and to improve access and the use of remote sensing, Earth observation, and geospatial data across the wider Pacific. However, most project‐driven initiatives are time limited; therefore, the lifespan of these initiatives depends on the interests and priorities of funding agencies (Jupiter, Mangubhai, et al., 2014; Lenz et al., 2022). Additionally, systems to support and operationalize remote sensing to inform decision‐making across the region are fragmented. Existing regional and national policies to support geospatial data management and remote sensing activities are not linked. Long‐term investment and interest in maintaining and developing remote sensing technical infrastructure, scientific research programs, and expert regional capacity across national agencies to support and sustain remote sensing across the wider Pacific are disparate.

**FIGURE 4:**
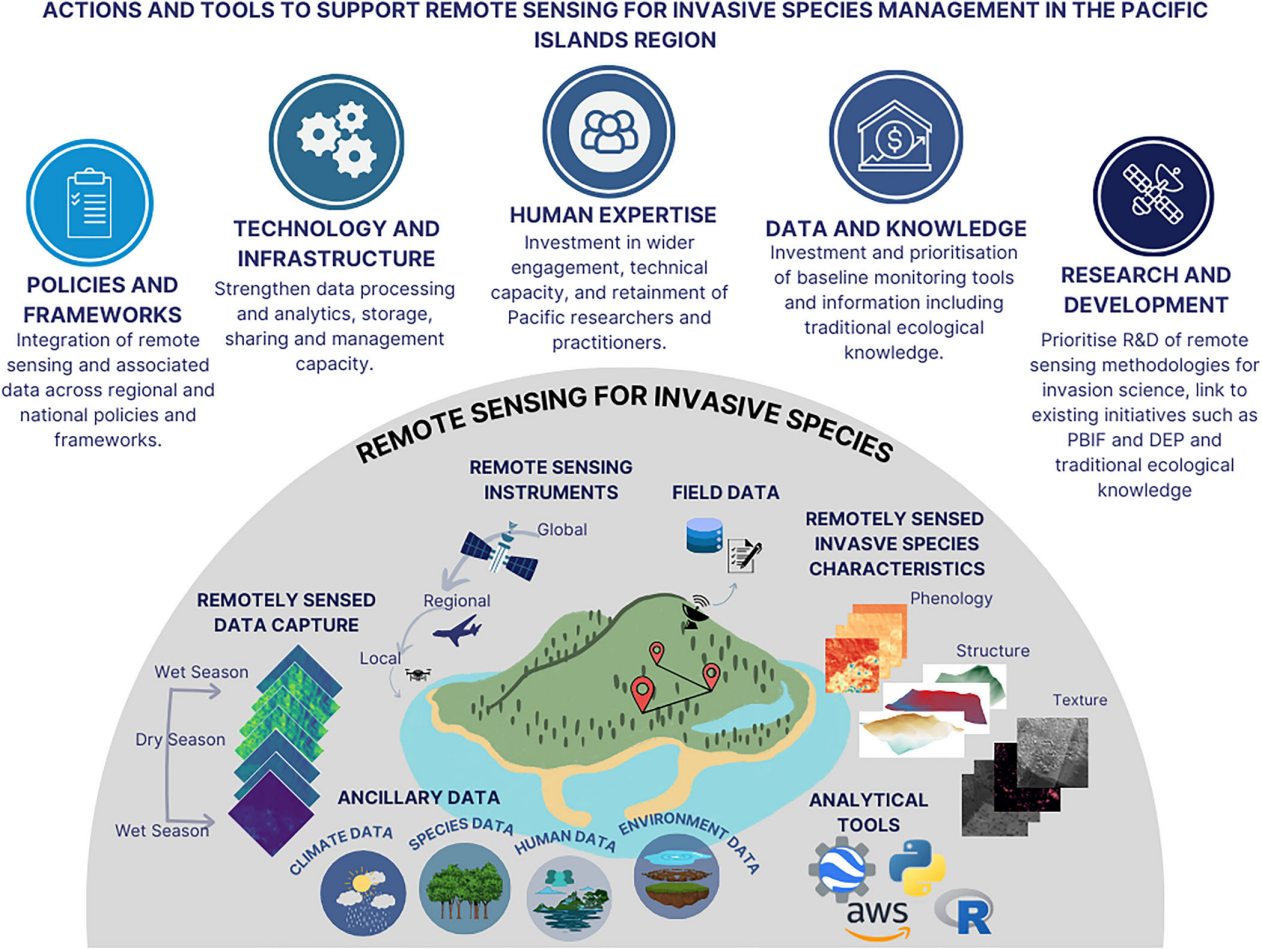
Actions and tools required to support remote sensing for invasive species management in the Pacific Islands region and facilitate an environment in which to implement remote sensing for research and as a source of information for decision‐making at various levels (PBIF, Pacific Biodiversity Information Facility; DEP, Digital Earth Pacific).

The available literature demonstrates a variety of methods and remotely sensed data that can be adapted, and fit for purpose, to implement in the Pacific Islands region, where invasions are widespread and require monitoring. Because regional invasive species activities are attracting donor funding and management attention, there exists an opportunity for remote sensing scientists and invasive species practitioners to collaborate. However, this will not be without its challenges, including organizational silos, which impede data sharing and management, limit expert capacity, and withhold technical infrastructure. For remote sensing to be operational, regional collaboration; dedicated funding; commitment to strengthen policies, frameworks, technology, and infrastructure; and development and retention of expert capacity are required (Figure 4). Our recommendations (below) are provided to support these 5 priority areas.

## RECOMMENDATIONS

### Integration of national policy frameworks

Vertical and horizontal integration of national policy frameworks must be prioritized to strengthen support for existing national systems. To support this integration, we suggest SPREP, as the lead regional environmental agency for invasive species management, conduct a regional gap analysis to assess the depth and breadth of each Pacific Island country's relevant legislation, plans, and policies that support the broad field of biodiversity and conservation. Where gaps exist, pathways to better integrate vertically and horizontally and strengthen existing policy frameworks should be identified. Vertical integration is the consistent alignment of nationally relevant legislation, plans, and policies with relevant international and regional policy frameworks, and horizontal integration aims to improve consistency of legislative instruments, plans, and policies across national‐level departments (Farrelly et al., 2021). These integrations improve coherence and coordination in implementation, thus increasing the potential to meet national targets (Farrelly et al., 2021). The gap analysis should make note of applicable global and regional policy frameworks and guiding documents that seek to improve invasive species management, including the *Guiding Framework for Invasive Species Management in the Pacific*, the *Global Strategy on Invasive Alien Species*, and those listed in Appendix S3.

### Inclusion of relevant geospatial decision‐support technology for long‐term monitoring

A crucial consideration for the gap analysis is the ability of legislation, plans, and policies to support the long‐term monitoring of invasive species by including relevant technologies, data, and systems. Geospatial and remote sensing data and methods, including DEP and PBIF and other regional technologies, should be explicitly included and supported in relevant local, national, and regional strategies and action plans, including the NISSAP and NBSAP. We suggest strategically linking these decision‐making tools to compliance mechanisms and regional indicators (e.g., site prioritization, biocontrol success) and incentives (e.g., results‐based payments and payments for ecosystem services) (SPREP, 2022). This would improve data availability and access, inform national and regional targets, including the *State of Environment and Conservation in the Pacific Islands* report, and strengthen technical infrastructure to sustain technologies, including remote sensing, that support decision‐making. This integration should be championed through regional networks, such as PILN and PIP, and regional agencies, such as SPREP and SPC.

### Design of competency‐based pathways to address gaps in technical expertise

To address gaps in technical expertise specific to each country, we recommend identifying regional practitioners, community leaders, technical advisers, researchers, and interested stakeholders across the region. This should include identification of experts in disciplines as diverse as information management, agriculture, biosecurity, remote sensing, Earth observation, natural resource management, and communications. This review could be conducted by PILN in collaboration with regional agencies, such as SPREP and SPC, and supported by funding from existing initiatives, such as PRISMSS.

The findings will help key stakeholders (e.g., tertiary education providers, e.g., USP), regional technical agencies (e.g., SPREP and SPC), regional partner organizations, and relevant technical advisers design national and regional competency‐based pathways to address national‐ and regional‐level gaps in expertise. Competency‐based pathways should be technically diverse and include short courses and degree programs and, where appropriate, incorporate regional initiatives, such as DEP and PBIF, and regional networks (i.e., PILN and PIP).

### Regional prioritization of Earth observation data

Invasive species and wide biodiversity decision‐support through Earth observation to Pacific Island countries must be regional priorities supported by SPREP and SPC. To facilitate these decision‐making services, long‐term funding to support infrastructure and programs, such as DEP, must be secured. Furthermore, regional participation in global networks, particularly the Group on Earth Observation Biodiversity Observation Network, would ensure global Earth observation data, technology, and infrastructure to deliver information to users are accessible and usable by even the Pacific Island countries with the least resources.

### Regional research and expertise through partnerships

Research partnerships may also help increase Pacific‐led research and build research capacity. Existing efforts include partnerships supported by SPREP and USP to expose early career conservation scientists to regional research. By partnering with the University of Newcastle (Australia) and New Zealand's Ministry of Foreign Affairs and Trade, for example, SPREP has secured several higher degree research scholarships in invasion science for Pacific Island students (University of Newcastle, 2019). C.C. is the recipient of such a scholarship. Pacific Island student engagement in regional conservation research is a successful model for meeting research and data needs and enhancing the capacity of the region.

### Data accessibility and management

Regional agencies, such as SPREP and SPC, are well positioned to support the collection and management of baseline information. Awareness education programs and engagement to share and maintain data, including species assessments, satellite imagery, and geospatial data, must reach a wider audience across multiple disciplines. This can be done through free and accessible courses, designed and conducted by SPREP and SPC, that link data accessibility and management to regional centralized data repositories, such as SPC's Pacific Data Hub and SPREP's INFORM portal. Targeted audiences should include regional networks involving conservation practitioners, such as PILN and PIP, and the wider geospatial community through the Pacific GIS and RS Council. Australia's Terrestrial Ecosystem Research Network offers a successful framework that can be adapted and transferred to the Pacific.

### Access to peer‐reviewed research and the latest science

To support peer‐to‐peer learning and knowledge curation, improving access to Pacific‐led peer‐reviewed research at the regional level is recommended. Access to resources, including the latest science, translations, and communication and publication support, could be facilitated at the regional level through the digital library services of SPREP and SPC and promoted through existing networks (e.g., PILN and PIP). Emphasis must be placed on publishing resources, including open‐source data and open‐access publications.

### Partnerships for establishing monitoring instruments

Long‐term monitoring tools are critical for invasive species management (Pacific Invasives Initiative, 2010). An independent regional review that evaluates current monitoring instruments and the status and type of biodiversity indicators that inform the regional and national state of environment reports should be conducted. In particular, the review should focus on available data, data usage, gaps, and accessibility, including the use of citizen science and traditional ecological knowledge.

Based on our review, relevant monitoring tools to improve baseline and long‐term monitoring data must be identified and collaboratively developed. This collaboration should be facilitated at the regional level, through PIP, SPREP, and SPC. Ideally, stakeholders would be members of local communities, national government agencies, domain experts, researchers, and regional and international organization representatives. With clearer objectives and effective instruments for monitoring and evaluation, countries will be better informed of best practice activities to manage invasive species and mitigate future threats.

## CONCLUSION

Invasion science and management at the regional and national levels are largely underfunded and underresearched in the Pacific Islands region. Conceptually, the application of remote sensing to inform invasive species management across Pacific countries should be well studied. However, persistent challenges are making it difficult to integrate remote sensing for decision‐making. These include the absence of any requirement to use remote sensing and geospatial data to support invasive species management in various national policies and strategies, disparate resourcing and technological infrastructure across the region, including internet accessibility and computing resources, and an absence of expert capacity in remote sensing, invasion science, and other related disciplines (e.g., ICT, communication and engagement, and management). We hope funders and regional actors will consider our recommendations. These include strengthening relevant local and regional policies, frameworks, and partnerships to improve monitoring and management, enhancing local and regional expert capacity and resources, and improving access to the latest scientific information and tools to better safeguard national and regional biodiversity.

## Supporting information

Supporting Information

## References

[cobi14344-bib-0001] Abeysinghe, T. , Simic Milas, A. , Arend, K. , Hohman, B. , Reil, P. , Gregory, A. , & Vázquez‐Ortega, A. (2019). Mapping invasive phragmites australis in the old woman creek estuary using UAV remote sensing and machine learning classifiers. Remote Sensing, 11(11), Article 1380.

[cobi14344-bib-0002] ADB (Asian Development Bank) . (2018). Tourism as a driver of growth in the Pacific (Issues in Pacific Development No. 2). https://www.adb.org/sites/default/files/publication/430171/tourism‐growth‐pacific.pdf

[cobi14344-bib-0003] Alvarez, S. , & Solis, D. (2018). Rapid response lowers eradication costs of invasive species: Evidence from Florida. Choices, 33(4), 1–9.

[cobi14344-bib-0004] Amani, M. , Ghorbanian, A. , Ahmadi, S. A. , Kakooei, M. , Moghimi, A. , Mirmazloumi, S. M. , Moghaddam, S. H. A. , Mahdavi, S. , Ghahremanloo, M. , Parsian, S. , Wu, Q. , & Brisco, B. (2020). Google Earth Engine cloud computing platform for remote sensing big data applications: A comprehensive review. IEEE Journal of Selected Topics in Applied Earth Observations and Remote Sensing, 13, 5326–5350.

[cobi14344-bib-0005] Arasumani, M. , Bunyan, M. , & Robin, V. V. (2021). Opportunities and challenges in using remote sensing for invasive tree species management, and in the identification of restoration sites in tropical montane grasslands. Journal of Environmental Management, 280, Article 111759. 33298397 10.1016/j.jenvman.2020.111759

[cobi14344-bib-0006] Asia Air Survey . (2014). Mapping Merremia peltata in Ole Pupu Pue National Park: Recommendations for ecological restoration (Forest Preservation Programme FY2010 in the Independent State of Samoa, Issue). Author.

[cobi14344-bib-0007] Asner, G. P. , Jones, M. O. , Martin, R. E. , Knapp, D. E. , & Hughes, R. F. (2008). Remote sensing of native and invasive species in Hawaiian forests. Remote Sensing of Environment, 112(5), 1912–1926.

[cobi14344-bib-0008] Atherton, J. (2015). Restoration plan consultation report for the OLPP NP. Secretariat of the Pacific Regional Environment Programme. https://brb.sprep.org/sites/default/files/2021‐12/restoration‐plan‐consultation‐report‐pupu‐pue‐national‐park.pdf

[cobi14344-bib-0009] Bäckstrand, K. (2006). Multi‐stakeholder partnerships for sustainable development: Rethinking legitimacy, accountability and effectiveness. European Environment, 16, 290–306.

[cobi14344-bib-0010] Baron, J. , & Hill, D. J. (2020). Monitoring grassland invasion by spotted knapweed (*Centaurea maculosa*) with RPAS‐acquired multispectral imagery. Remote Sensing of Environment, 249, Article 112008.

[cobi14344-bib-0011] Bellard, C. , Cassey, P. , & Blackburn, T. M. (2016). Alien species as a driver of recent extinctions. Biology Letters, 12(2), Article 20150623. 10.1098/rsbl.2015.0623 26888913 PMC4780541

[cobi14344-bib-0012] Bolch, E. A. , Santos, M. J. , Ade, C. , Khanna, S. , Basinger, N. T. , Reader, M. O. , & Hestir, E. L. (2020). Remote detection of invasive alien species. In J. Cavender‐Bares , J. A. Gamon , & P. A. Townsend (Eds.), Remote sensing of plant biodiversity (pp. 267–307). Springer International Publishing.

[cobi14344-bib-0013] Bonte‐Grapentin, M. , Meier, P. , & Saito, K. (2017). Lessons from mapping geeks: How aerial technology is helping Pacific Island countries recover from natural disasters. World Bank. https://blogs.worldbank.org/eastasiapacific/lessons‐mapping‐geeks‐how‐aerial‐technology‐helping‐pacific‐island‐countries‐recover‐natural

[cobi14344-bib-0014] Boyte, S. P. , Wylie, B. K. , & Major, D. J. (2015). Mapping and monitoring cheatgrass dieoff in rangelands of the Northern Great Basin, USA. Rangeland Ecology & Management, 68(1), 18–28. 10.1016/j.rama.2014.12.005

[cobi14344-bib-0015] Boyte, S. P. , Wylie, B. K. , Major, D. J. , & Brown, J. F. (2015). The integration of geophysical and enhanced Moderate Resolution Imaging Spectroradiometer Normalized Difference Vegetation Index data into a rule‐based, piecewise regression‐tree model to estimate cheatgrass beginning of spring growth. International Journal of Digital Earth, 8(2), 118–132.

[cobi14344-bib-0016] Bradley, B. A. (2014). Remote detection of invasive plants: A review of spectral, textural and phenological approaches. Biological Invasions, 16, 1411–1425.

[cobi14344-bib-0017] Bradley, B. A. , Curtis, C. A. , Fusco, E. J. , Abatzoglou, J. T. , Balch, J. K. , Dadashi, S. , & Tuanmu, M.‐N. (2018). Cheatgrass (*Bromus tectorum*) distribution in the intermountain Western United States and its relationship to fire frequency, seasonality, and ignitions. Biological Invasions, 20(6), 1493–1506.

[cobi14344-bib-0018] Bradley, B. A. , & Mustard, J. F. (2005). Identifying land cover variability distinct from land cover change: Cheatgrass in the Great Basin. Remote Sensing of Environment, 94(2), 204–213.

[cobi14344-bib-0019] Bransky, N. , Sankey, T. , Sankey, J. B. , Johnson, M. , & Jamison, L. (2021). Monitoring Tamarix changes using WorldView‐2 satellite imagery in Grand Canyon National Park, Arizona. Remote Sensing, 13(5), Article 958. 10.3390/rs13050958

[cobi14344-bib-0020] Brewer, K. , Lottering, R. , & Peerbhay, K. (2022). Remote sensing of invasive alien wattle using image texture ratios in the low‐lying Midlands of KwaZulu‐Natal, South Africa. Remote Sensing Applications: Society and Environment, 26, Article 100769.

[cobi14344-bib-0021] Brewington, L. , Eichelberger, B. , Read, N. , Parsons, E. , Kerkering, H. , Martin, C. , Miles, W. , Idechong, J. , & Burgett, J. (2023). Pacific Island perspectives on invasive species and climate change. In S. J. Walsh , C. F. Mena , J. R. Stewart , & J. P. Muñoz Pérez (Eds.), Island ecosystems: Challenges to sustainability (pp. 59–78). Springer International Publishing.

[cobi14344-bib-0022] Britton, J. (2000). GIS capacity building in the Pacific Island countries: Facing the realities of technology, resources, geography and cultural difference. Cartographica: The International Journal for Geographic Information and Geovisualization, 37(4), 7–19.

[cobi14344-bib-0023] Brock, K. C. , & Daehler, C. C. (2021). Plant naturalization trends reflect socioeconomic history and show a high likelihood of inter‐island spread in Hawaiʻi. Invasive Plant Science and Management, 14(3), 135–146.

[cobi14344-bib-0024] Brodie, G. , Pikacha, P. , & Tuiwawa, M. (2013). Biodiversity and conservation in the Pacific Islands: Why are we not succeeding? In P. H. Raven , N. S. Sodhi , & L. Gibson (Eds.), Conservation biology: Voices from the tropics (pp. 181–187). John Wiley & Sons. 10.1002/9781118679838.ch21

[cobi14344-bib-0025] Convention on Biological Diversity (CBD) . (2022). Kunming‐Montreal Global Biodiversity Framework . https://www.cbd.int/doc/decisions/cop‐15/cop‐15‐dec‐04‐en.pdf

[cobi14344-bib-0026] Chance, C. M. , Coops, N. C. , Plowright, A. A. , Tooke, T. R. , Christen, A. , & Aven, N. (2016). Invasive shrub mapping in an urban environment from hyperspectral and LiDAR‐derived attributes. Frontiers in Plant Science, 7, Article 1528. 10.3389/fpls.2016.01528 27818664 PMC5073150

[cobi14344-bib-0027] Chen, S. , Zhang, R. , Su, H. , Tian, J. , & Xia, J. (2010). SAR and multispectral image fusion using generalized IHS transform based on trous wavelet and EMD decompositions. IEEE Sensors Journal, 10(3), 737–745. 10.1109/JSEN.2009.2038661

[cobi14344-bib-0028] Chetty, S. , Mutanga, O. , & Lottering, R. (2021). Detecting and mapping invasive *Parthenium hysterophorus* L. along the northern coastal belt of KwaZulu‐Natal, South Africa using image texture. Scientific African, 13, Article e00966.

[cobi14344-bib-0029] Chiang, S.‐H. , & Valdez, M. (2019). Tree species classification by integrating satellite imagery and topographic variables using maximum entropy method in a Mongolian forest. Forests, 10(11), Article 961.

[cobi14344-bib-0030] CRCSI (The Australia and New Zealand Cooperative Research Centre for Spatial Information) . (2017). Volume 1B: Data—Image interpretation (Earth observation: Data, processing and applications). Australia and New Zealand Cooperative Research Centre for Spatial Information. https://www.crcsi.com.au/assets/Consultancy‐Reports‐and‐Case‐Studies/Earth‐Observation‐reports‐updated‐Feb‐2019/Vol1B‐low‐res‐24MB.pdf

[cobi14344-bib-0031] Dahal, D. , Pastick, N. J. , Boyte, S. P. , Parajuli, S. , Oimoen, M. J. , & Megard, L. J. (2022). Multi‐species inference of exotic annual and native perennial grasses in rangelands of the Western United States using harmonized Landsat and Sentinel‐2 data. Remote Sensing, 14(4), Article 807.

[cobi14344-bib-0032] Dai, J. , Roberts, D. , Stow, D. , An, L. , Hall, S. , Yabiku, S. , & Kyriakidis, P. (2020). Mapping understory invasive plant species with field and remotely sensed data in Chitwan, Nepal. Remote Sensing of Environment, 250, Article 112037.

[cobi14344-bib-0033] Dash, J. P. , Watt, M. S. , Paul, T. S. H. , Morgenroth, J. , & Pearse, G. D. (2019). Early detection of invasive exotic trees using UAV and manned aircraft multispectral and LiDAR data. Remote Sensing, 11(15), Article 1812.

[cobi14344-bib-0034] Davis, B. (1993). GIS facilities at USP. Fiji User Group GIS and Remote Sensing News, 1(9301), 1–2. https://nxga6f.n3cdn1.secureserver.net/wp‐content/uploads/2023/07/9301.pdf

[cobi14344-bib-0035] Day, M. D. , & Winston, R. L. (2016). Biological control of weeds in the 22 Pacific Island countries and territories: Current status and future prospects. NeoBiota, 30, 167–192.

[cobi14344-bib-0036] Denslow, J. S. (2008). Invasions and impacts of exotic plants in the Pacific Islands. In R. D. van Klinken , V. A. Osten , F. D. Panetta , & J. C. Scanlan (Eds.), Proceedings of the 16th Australian Weeds Conference, Brisbane (pp. 14–16). Queensland Weeds Society.

[cobi14344-bib-0037] Deur, M. , Gašparović, M. , & Balenović, I. (2020). Tree species classification in mixed deciduous forests using very high spatial resolution satellite imagery and machine learning methods. Remote Sensing, 12(23), Article 3926. 10.3390/rs12233926

[cobi14344-bib-0038] Diagne, C. , Leroy, B. , Vaissière, A.‐C. , Gozlan, R. E. , Roiz, D. , Jarić, I. , Salles, J.‐M. , Bradshaw, C. J. A. , & Courchamp, F. (2021). High and rising economic costs of biological invasions worldwide. Nature, 592(7855), 571–576. 33790468 10.1038/s41586-021-03405-6

[cobi14344-bib-0039] Domingo, D. , Pérez‐Rodríguez, F. , Gómez‐García, E. , & Rodríguez‐Puerta, F. (2023). Assessing the efficacy of phenological spectral differences to detect invasive alien *Acacia dealbata* using Sentinel‐2 data in Southern Europe. Remote Sensing, 15(3), Article 722.

[cobi14344-bib-0040] Dovey, L. , Orapa, W. , Randall, S. , Cullen, J. M. , Briese, D. T. , Kriticos, D. J. , Lonsdale, W. M. , Morin, L. , & Scott, J. K. (2004). The need to build biological control capacity in the Pacific. In J. M. Cullen , D. T. Briese , D. J. Kriticos , W. M. Lonsdale , L. Morin , & J. K. Scott (Eds.), Proceedings of the XI International Symposium on Biological Control of Weeds (pp. 36–41). CSIRO Entomology.

[cobi14344-bib-0041] Dronova, I. , Spotswood, E. N. , & Suding, K. N. (2017). Opportunities and constraints in characterizing landscape distribution of an invasive grass from very high resolution multi‐spectral imagery. Frontiers in Plant Science, 8, Article 890. 10.3389/fpls.2017.00890 28611806 PMC5447865

[cobi14344-bib-0042] Dronova, I. , & Taddeo, S. (2022). Remote sensing of phenology: Towards the comprehensive indicators of plant community dynamics from species to regional scales. Journal of Ecology, 110(7), 1460–1484.

[cobi14344-bib-0043] Elkind, K. , Sankey, T. T. , Munson, S. M. , & Aslan, C. E. (2019). Invasive buffelgrass detection using high‐resolution satellite and UAV imagery on Google Earth Engine. Remote Sensing in Ecology and Conservation, 5(4), 318–331.

[cobi14344-bib-0044] Fantle‐Lepczyk, J. E. , Haubrock, P. J. , Kramer, A. M. , Cuthbert, R. N. , Turbelin, A. J. , Crystal‐Ornelas, R. , Diagne, C. , & Courchamp, F. (2022). Economic costs of biological invasions in the United States. Science of The Total Environment, 806(3), Article 151318. 34743879 10.1016/j.scitotenv.2021.151318

[cobi14344-bib-0045] Farrelly, T. A. , Borrelle, S. B. , & Fuller, S. (2021). The strengths and weaknesses of Pacific Islands plastic pollution policy frameworks. Sustainability, 13(3), Article 1252.

[cobi14344-bib-0046] Global Biodiversity Information Facility (GBIF) . (2024). Global Core Biodata Resource . https://www.gbif.org/

[cobi14344-bib-0047] Group on Earth Observations (GEO) . (2019). Earth observation cooperation in the Pacific: Talanoa outcome statement . https://earthobservations.org/documents/geo16/cooperation_in_the_pacific_talanoa_outcome_statement.pdf

[cobi14344-bib-0048] Ghulam, A. , Porton, I. , & Freeman, K. (2014). Detecting subcanopy invasive plant species in tropical rainforest by integrating optical and microwave (InSAR/PolInSAR) remote sensing data, and a decision tree algorithm. ISPRS Journal of Photogrammetry and Remote Sensing, 88, 174–192.

[cobi14344-bib-0049] Goldfarb, L. (2019). UAVs and trees: How American Samoa is using drones to track invasive species and monitor forest health. Western Forestry Leadership Coalition. https://www.thewflc.org/news/blog/uavs‐and‐trees‐how‐american‐samoa‐using‐drones‐track‐invasive‐species‐and‐monitor‐forest

[cobi14344-bib-0050] Government of Fiji . (2020). National Biodiversity Strategy and Action Plan for Fiji 2020–2025. Department of Environment. https://www.mowe.gov.fj/wp‐content/uploads/2020/06/National‐Biodiversity‐Strategy‐Action‐Plan.pdf

[cobi14344-bib-0051] Government of Niue . (2012). Niue's National Invasive Species Strategy and Action Plan 2013–2020 . https://faolex.fao.org/docs/pdf/niu176159.pdf

[cobi14344-bib-0052] Government of Niue . (2015). Niue National Biodiversity Strategy and Action Plan. Bateson Publishing Limited. https://www.cbd.int/doc/world/nu/nu‐nbsap‐v2‐en.pdf

[cobi14344-bib-0053] Government of Samoa . (2019). Samoa National Invasive Species Strategy and Action Plan (NISSAP): 2019–2024. Ministry of Natural Resources and Environment. https://www.sprep.org/attachments/VirLib/Regional/nissap‐samoa‐2019‐2024.pdf

[cobi14344-bib-0054] Government of Solomon Islands . (2016). The National Biodiversity Strategic Action Plan . https://www.cbd.int/doc/world/sb/sb‐nbsap‐v2‐en.pdf

[cobi14344-bib-0055] Groom, Q. , Adriaens, T. , Desmet, P. , Simpson, A. , De Wever, A. , Bazos, I. , Cardoso, A. , Charles, L. , Christopoulou, A. , Gazda, A. , Helmisaari, H. , Hobern, D. , Josefsson, M. , Lucy, F. , Dragana, M. , Oszako, T. , Pergl, J. , Petrović‐Obradović, O. , Prévot, C. , & Vanderhoeven, S. (2017). Seven recommendations to make your invasive alien species data more useful. Frontiers in Applied Mathematics and Statistics, 3, Article 13. 10.3389/fams.2017.00013

[cobi14344-bib-0056] Hanley, N. , & Roberts, M. (2019). The economic benefits of invasive species management. People and Nature, 1(2), 124–137.

[cobi14344-bib-0057] Hauglin, M. , & Ørka, H. O. (2016). Discriminating between Native Norway Spruce and Invasive Sitka Spruce—A comparison of multitemporal Landsat 8 imagery, aerial images and airborne laser scanner data. Remote Sensing, 8(5), Article 363.

[cobi14344-bib-0058] He, K. S. , Rocchini, D. , Neteler, M. , & Nagendra, H. (2011). Benefits of hyperspectral remote sensing for tracking plant invasions. Diversity and Distributions, 17(3), 381–392.

[cobi14344-bib-0059] Howey, M. , Sullivan, F. , Brouwer Burg, M. , & Palace, M. (2020). Remotely sensed big data and iterative approaches to cultural feature detection and past landscape process analysis. Journal of Field Archaeology, 45, S27–S38. 10.1080/00934690.2020.1713435

[cobi14344-bib-0060] Huang, C.‐y. , & Asner, G. P. (2009). Applications of remote sensing to alien invasive plant studies. Sensors, 9(6), 4869–4889. 22408558 10.3390/s90604869 PMC3291943

[cobi14344-bib-0061] International Union for Conservation of Nature (IUCN) . (2000). IUCN guidelines for the prevention of biodiversity loss caused by alien invasive species . https://portals.iucn.org/library/node/12413

[cobi14344-bib-0062] Jupiter, S. D. , Jenkins, A. P. , Long, W. , Maxwell, S. L. , Carruthers, T. J. B. , Hodge, K. B. , Tamelander, J. , Govan, H. , & Watson, J. E. M. (2014). Principles for integrated island management in the tropical Pacific. Pacific Conservation Biology, 20(2), 193–205.

[cobi14344-bib-0063] Jupiter, S. D. , Mangubhai, S. , & Kingsford, R. (2014). Conservation of biodiversity in the Pacific Islands of Oceania: Challenges and opportunities. Pacific Conservation Biology, 20(2), 206–220.

[cobi14344-bib-0064] Kattenborn, T. , Lopatin, J. , Förster, M. , Braun, A. C. , & Fassnacht, F. E. (2019). UAV data as alternative to field sampling to map woody invasive species based on combined Sentinel‐1 and Sentinel‐2 data. Remote Sensing of Environment, 227, 61–73.

[cobi14344-bib-0065] Kearney, S. G. , Carwardine, J. , Reside, A. E. , Fisher, D. O. , Maron, M. , Doherty, T. S. , Legge, S. , Silcock, J. , Woinarski, J. C. Z. , Garnett, S. T. , Wintle, B. A. , & Watson, J. E. M. (2019). The threats to Australia's imperilled species and implications for a national conservation response. Pacific Conservation Biology, 25(3), 231–244.

[cobi14344-bib-0066] Keppel, G. , Morrison, C. , Meyer, J.‐Y. , & Boehmer, H. J. (2014). Isolated and vulnerable: The history and future of Pacific Island terrestrial biodiversity. Pacific Conservation Biology, 20(2), 136–145.

[cobi14344-bib-0067] Keppel, G. , Morrison, C. , Watling, D. , Tuiwawa, M. , & Rounds, I. (2012). Conservation in tropical Pacific Island countries: Why most current approaches are failing. Conservation Letters, 5(4), 256–265.

[cobi14344-bib-0068] Keppel, G. , Peters, S. , Taoi, J. , Raituku, N. , & Thomas‐Moko, N. (2021). The threat by the invasive African tulip tree, *Spathodea campanulata* P.Beauv., for the critically endangered Fijian tree, *Pterocymbium oceanicum* A.C.Sm.; revisiting an assessment based on expert knowledge after extensive field surveys. Pacific Conservation Biology, 28(2), 164–173.

[cobi14344-bib-0069] Koerner, L. M. , Chadwick, M. A. , & Tebbs, E. J. (2022). Mapping invasive strawberry guava (*Psidium cattleianum*) in tropical forests of Mauritius with Sentinel‐2 and machine learning. International Journal of Remote Sensing, 43(3), 841–872.

[cobi14344-bib-0070] Laba, M. , Blair, B. , Downs, R. , Monger, B. , Philpot, W. , Smith, S. , Sullivan, P. , & Baveye, P. C. (2010). Use of textural measurements to map invasive wetland plants in the Hudson River National Estuarine Research Reserve with IKONOS satellite imagery. Remote Sensing of Environment, 114(4), 876–886.

[cobi14344-bib-0071] Labonté, J. , Drolet, G. , Sylvain, J.‐D. , Thiffault, N. , Hébert, F. , & Girard, F. (2020). Phenology‐based mapping of an alien invasive species using time series of multispectral satellite data: A case‐study with glossy buckthorn in Québec, Canada. Remote Sensing, 12(6), Article 922.

[cobi14344-bib-0072] Laginhas, B. B. , Fertakos, M. E. , & Bradley, B. A. (2023). We don't know what we're missing: Evidence of a vastly undersampled invasive plant pool. Ecological Applications, 33(2), Article e2776. 36315354 10.1002/eap.2776

[cobi14344-bib-0073] Lake, T. A. , Briscoe Runquist, R. D. , & Moeller, D. A. (2022). Deep learning detects invasive plant species across complex landscapes using Worldview‐2 and Planetscope satellite imagery. Remote Sensing in Ecology and Conservation, 8(6), 875–899.

[cobi14344-bib-0074] Lenz, M. I. , Galvin, S. , Keppel, G. , Gopaul, S. , Kowasch, M. , Dyer, M. J. , Watling, D. , Lodhar, S. Y. F. , Hanson, G. C. , Erasmi, S. , & Boehmer, H. J. (2022). Where to invade next: Inaction on biological invasions threatens sustainability in a small island developing state of the tropical South Pacific. In P. S. Low (Ed.), Sustainable development: Asia‐Pacific perspectives (pp. 393–406). Cambridge University Press. 10.1017/9780511977961.035

[cobi14344-bib-0075] Liang, W. , Abidi, M. , Carrasco, L. , McNelis, J. , Tran, L. , Li, Y. , & Grant, J. (2020). Mapping vegetation at species level with high‐resolution multispectral and lidar data over a large spatial area: A case study with Kudzu. Remote Sensing, 12(4), Article 609.

[cobi14344-bib-0076] Löfstedt, T. , Brynolfsson, P. , Asklund, T. , Nyholm, T. , & Garpebring, A. (2019). Gray‐level invariant Haralick texture features. PLoS ONE, 14(2), Article e0212110. 30794577 10.1371/journal.pone.0212110 PMC6386443

[cobi14344-bib-0077] Lopatin, J. , Dolos, K. , Kattenborn, T. , & Fassnacht, F. E. (2019). How canopy shadow affects invasive plant species classification in high spatial resolution remote sensing. Remote Sensing in Ecology and Conservation, 5(4), 302–317.

[cobi14344-bib-0078] Lottering, R. , Mutanga, O. , Peerbhay, K. , & Ismail, R. (2019). Detecting and mapping Gonipterus scutellatus induced vegetation defoliation using WorldView‐2 pan‐sharpened image texture combinations and an artificial neural network. Journal of Applied Remote Sensing, 13(1), Article 014513.

[cobi14344-bib-0079] Lottering, R. T. , Govender, M. , Peerbhay, K. , & Lottering, S. (2020). Comparing partial least squares (PLS) discriminant analysis and sparse PLS discriminant analysis in detecting and mapping *Solanum mauritianum* in commercial forest plantations using image texture. ISPRS Journal of Photogrammetry and Remote Sensing, 159, 271–280.

[cobi14344-bib-0080] Lowry, B. J. , Lowry, J. H. , Jarvis, K. J. , Keppel, G. , Thaman, R. R. , & Boehmer, H. J. (2020). Spatial patterns of presence, abundance, and richness of invasive woody plants in relation to urbanization in a tropical island setting. Urban Forestry & Urban Greening, 48, Article 126516.

[cobi14344-bib-0081] Marcinkowska‐Ochtyra, A. , Jarocińska, A. , Bzdęga, K. , & Tokarska‐Guzik, B. (2018). Classification of expansive grassland species in different growth stages based on hyperspectral and LiDAR data. Remote Sensing, 10(12), Article 2019. 10.3390/rs10122019

[cobi14344-bib-0082] Marzialetti, F. , Frate, L. , De Simone, W. , Frattaroli, A. R. , Acosta, A. T. , & Carranza, M. L. (2021). Unmanned aerial vehicle (UAV)‐based mapping of *Acacia saligna* invasion in the Mediterranean Coast. Remote Sensing, 13(17), Article 3361.

[cobi14344-bib-0083] McGrannachan, C. , Mitchell, C. , & Probst, C. (2021). Feasibility of biological control of taro vine, Epipremnum pinnatum (L.) Engl. cv. Aureum in the Pacific region (Contract Report LC4015 for the Ministry of Foreign Affairs and Trade). Manaaki Whenua—Landcare Research.

[cobi14344-bib-0084] Meyer, J.‐Y. (2014). Critical issues and new challenges for research and management of invasive plants in the Pacific Islands. Pacific Conservation Biology, 20(2), 146–164.

[cobi14344-bib-0085] Meyer, J.‐Y. , Pouteau, R. , Spotswood, E. , Taputuarai, R. , & Fourdrigniez, M. (2015). The importance of novel and hybrid habitats for plant conservation on islands: A case study from Moorea (South Pacific). Biodiversity and Conservation, 24(1), 83–101.

[cobi14344-bib-0086] Meyer, S. E. , Callaham, M. A., Jr. , Stewart, J. E. , & Warren, S. D. (2021). Invasive species response to natural and anthropogenic disturbance. In T. M. Poland , T. Patel‐Weynand , D. M. L. Finch , C. F. Miniat , D. C. Hayes , & V. M. Lopez (Eds.), Invasive species in forests and rangelands of the United States: A comprehensive science synthesis for the United States forest sector (pp. 119–144). Springer.

[cobi14344-bib-0087] Mielczarek, D. , Sikorski, P. , Archiciński, P. , Ciężkowski, W. , Zaniewska, E. , & Chormański, J. (2023). The use of an airborne laser scanner for rapid identification of invasive tree species *Acer negundo* in riparian forests. Remote Sensing, 15(1), Article 212.

[cobi14344-bib-0088] Montero, D. , Aybar, C. , Mahecha, M. D. , Martinuzzi, F. , Söchting, M. , & Wieneke, S. (2023). A standardized catalogue of spectral indices to advance the use of remote sensing in Earth system research. Scientific Data, 10(1), Article 197. 37031236 10.1038/s41597-023-02096-0 PMC10082855

[cobi14344-bib-0089] Newbold, T. , Hudson, L. N. , Hill, S. L. L. , Contu, S. , Lysenko, I. , Senior, R. A. , Börger, L. , Bennett, D. J. , Choimes, A. , Collen, B. , Day, J. , De Palma, A. , Díaz, S. , Echeverria‐Londoño, S. , Edgar, M. J. , Feldman, A. , Garon, M. , Harrison, M. L. K. , Alhusseini, T. , … Purvis, A. (2015). Global effects of land use on local terrestrial biodiversity. Nature, 520(7545), 45–50. 25832402 10.1038/nature14324

[cobi14344-bib-0090] Niphadkar, M. , & Nagendra, H. (2016). Remote sensing of invasive plants: Incorporating functional traits into the picture. International Journal of Remote Sensing, 37(13), 3074–3085.

[cobi14344-bib-0091] Nunn, P. D. , Kumar, L. , Eliot, I. , & McLean, R. F. (2016). Classifying Pacific islands. Geoscience Letters, 3, Article 7. 10.1186/s40562-016-0041-8

[cobi14344-bib-0092] Pacific Invasives Initiative . (2010). Invasive Species Management in the Pacific: A review on National Plans and Current Activities. Author.

[cobi14344-bib-0093] Parker, K. , Elmes, A. , Boucher, P. , Hallett, R. A. , Thompson, J. E. , Simek, Z. , Bowers, J. , & Reinmann, A. B. (2021). Crossing the great divide: Bridging the researcher–practitioner gap to maximize the utility of remote sensing for invasive species monitoring and management. Remote Sensing, 13(20), Article 4142.

[cobi14344-bib-0094] Paynter, Q. , Harman, H. , & Waipara, N. (2006). Prospects for biological control of Merremia peltata (Contract Report LC0506/177 for Conservation International, in partnership with The Pacific Invasives Initiative). Manaaki Whenua—Landcare Research. http://www.botany.hawaii.edu/basch/uhnpscesu/pdfs/sam/Paynter2006AS.pdf

[cobi14344-bib-0095] Paz‐Kagan, T. , Silver, M. , Panov, N. , & Karnieli, A. (2019). Multispectral approach for identifying invasive plant species based on flowering phenology characteristics. Remote Sensing, 11(8), Article 953. 10.3390/rs11080953

[cobi14344-bib-0096] Pene, C. (2006). Integrating urban spatial data using Geographic Information Systems. University of the South Pacific.

[cobi14344-bib-0097] Poidevin, M. (1995). Stratification of secondary forest in Fiji. GIS & Remote Sensing News, 10(9501), 8. https://nxga6f.n3cdn1.secureserver.net/wp‐content/uploads/2023/07/9501.pdf

[cobi14344-bib-0098] Pouteau, R. , Meyer, J.‐Y. , Fourdrigniez, M. , & Taputuarai, R. (Eds.). (2013). Novel ecosystems in the Pacific Islands: Assessing loss, fragmentation and alteration of native forests by invasive alien plants on the island of Moorea (French Polynesia). Presses Universitaires de Provence & The Australian National University e‐Press.

[cobi14344-bib-0099] Pyšek, P. , Hulme, P. E. , Simberloff, D. , Bacher, S. , Blackburn, T. M. , Carlton, J. T. , Dawson, W. , Essl, F. , Foxcroft, L. C. , Genovesi, P. , Jeschke, J. M. , Kühn, I. , Liebhold, A. M. , Mandrak, N. E. , Meyerson, L. A. , Pauchard, A. , Pergl, J. , Roy, H. E. , Seebens, H. , … Richardson, D. M. (2020). Scientists' warning on invasive alien species. Biological Reviews, 95(6), 1511–1534. 32588508 10.1111/brv.12627 PMC7687187

[cobi14344-bib-0100] Reaser, J. K. , Meyerson, L. A. , Cronk, Q. , De Poorter, M. A. J. , Eldrege, L. G. , Green, E. , Kairo, M. , Latasi, P. , Mack, R. N. , Mauremootoo, J. , O'Dowd, D. , Orapa, W. , Sastroutomo, S. , Saunders, A. , Shine, C. , Thrainsson, S. , & Vaiutu, L. (2007). Ecological and socioeconomic impacts of invasive alien species in island ecosystems. Environmental Conservation, 34(2), 98–111.

[cobi14344-bib-0101] Russell, J. C. , Meyer, J.‐Y. , Holmes, N. D. , & Pagad, S. (2017). Invasive alien species on islands: Impacts, distribution, interactions and management. Environmental Conservation, 44(4), 359–370.

[cobi14344-bib-0102] Sabat‐Tomala, A. , Raczko, E. , & Zagajewski, B. (2020). Comparison of support vector machine and random forest algorithms for invasive and expansive species classification using airborne hyperspectral data. Remote Sensing, 12(3), Article 516.

[cobi14344-bib-0103] Sakai, A. K. , Allendorf, F. W. , Holt, J. S. , Lodge, D. M. , Molofsky, J. , With, K. A. , Baughman, S. , Cabin, R. J. , Cohen, J. E. , Ellstrand, N. C. , McCauley, D. E. , O'Neil, P. , Parker, I. M. , Thompson, J. N. , & Weller, S. G. (2001). The population biology of invasive species. Annual Review of Ecology and Systematics, 32(1), 305–332.

[cobi14344-bib-0104] Seebens, H. , Blackburn, T. M. , Dyer, E. E. , Genovesi, P. , Hulme, P. E. , Jeschke, J. M. , Pagad, S. , Pyšek, P. , Winter, M. , Arianoutsou, M. , Bacher, S. , Blasius, B. , Brundu, G. , Capinha, C. , Celesti‐Grapow, L. , Dawson, W. , Dullinger, S. , Fuentes, N. , Jäger, H. , … Essl, F. (2017). No saturation in the accumulation of alien species worldwide. Nature Communications, 8(1), Article 14435. 10.1038/ncomms14435 PMC5316856 28198420

[cobi14344-bib-0105] Seebens, H. , Essl, F. , Hulme, P. E. , & van Kleunen, M. (2022). Development of pathways of global plant invasions in space and time. In D. R. Clements , M. K. Upadhyaya , S. Joshi , & A. Shrestha (Eds.), Global plant invasions (pp. 53–69). Springer International Publishing. 10.1007/978-3-030-89684-3_3

[cobi14344-bib-0106] Shendryk, Y. , Rossiter‐Rachor, N. A. , Setterfield, S. A. , & Levick, S. R. (2020). Leveraging high‐resolution satellite imagery and gradient boosting for invasive weed mapping. IEEE Journal of Selected Topics in Applied Earth Observations and Remote Sensing, 13, 4443–4450.

[cobi14344-bib-0107] Singh, K. K. , Davis, A. J. , & Meentemeyer, R. K. (2015). Detecting understory plant invasion in urban forests using LiDAR. International Journal of Applied Earth Observation and Geoinformation, 38, 267–279.

[cobi14344-bib-0108] Skarpaas, O. , & Shea, K. (2007). Dispersal patterns, dispersal mechanisms, and invasion wave speeds for invasive thistles. The American Naturalist, 170(3), 421–430. 10.1086/519854 17879192

[cobi14344-bib-0109] SPC (Pacific Community) . (2019). Pacific Earth Observation Coordination Meeting. Pacific Community. https://spccfpstore1.blob.core.windows.net/digitallibrary‐docs/files/b2/b2ac2fd9ef1d526d14f9bcd82a7b4c83.pdf?sv=2015‐12‐11&sr=b&sig=gB5dRPRfZ3CzcMS%2BCR9y%2FG7%2FAV4QlH1FDN6yhxmm6g4%3D&se=2024‐12‐22T02%3A56%3A54Z&sp=r&rscc=public%2C%20max‐age%3D864000%2C%20max‐stale%3D86400&rsct=application%2Fpdf&rscd=inline%3B%20filename%3D%22Pacific_Earth_Observation_Coordination_Meeting_2019_Meeting_Report.pdf%22

[cobi14344-bib-0110] SPC . (2021). Digital Earth Pacific: Needs assessment report . Pacific Community. https://spccfpstore1.blob.core.windows.net/digitallibrary‐docs/files/a1/a1a4e04fbb05db15e8502de12407a9f2.pdf?sv=2015‐12‐11&sr=b&sig=jy8jDQAsDxZAWZTheYXw%2BnzhA3ohXwalCPLGHPE%2BS5g%3D&se=2024‐12‐22T02%3A34%3A04Z&sp=r&rscc=public%2C%20max‐age%3D864000%2C%20max‐stale%3D86400&rsct=application%2Fpdf&rscd=inline%3B%20filename%3D%22Digital_Earth_Pacific_needs_assessment_report_web.pdf%22

[cobi14344-bib-0111] SPC . (2022). Mapping our pacific geospatial future. Pacific Community. https://www.spc.int/updates/blog/2022/06/mapping‐our‐pacific‐geospatial‐future

[cobi14344-bib-0112] SPC . (2023). Pacific Islands Pest List Database. Pacific Community.

[cobi14344-bib-0142] Secretariat of the Pacific Regional Environment Programme (SPREP) . (2000). Invasive species in the Pacific: A technical review and draft regional strategy . https://www.sprep.org/att/publication/000159_Invasive_strategy_and_species.pdf

[cobi14344-bib-0113] Secretariat of the Pacific Regional Environment Programme (SPREP) . (2009). Guidelines for invasive species management in the Pacific: A Pacific strategy for managing pests, weeds and other invasive species . Secretariat of the Pacific Regional Environment Programme. https://www.sprep.org/att/publication/000699_RISSFinalLR.pdf

[cobi14344-bib-0114] Secretariat of the Pacific Regional Environment Programme (SPREP) . (2016). Battling invasive species in the Pacific: Outcomes of the regional GEF‐PAS IAS project . Secretariat of the Pacific Regional Environment Programme. https://www.sprep.org/attachments/Publications/BEM/battling‐invasive‐species‐pacific.pdf

[cobi14344-bib-0115] Secretariat of the Pacific Regional Environment Programme (SPREP) . (2020a). State of environment and conservation in the Pacific Islands: 2020 Regional Report . Secretariat of the Pacific Regional Environment Programme. https://library.sprep.org/sites/default/files/2021‐03/SOE‐conservation‐pacific‐regional‐report.pdf

[cobi14344-bib-0116] Secretariat of the Pacific Regional Environment Programme (SPREP) . (2020b). Use natural enemies to manage widespread weeds in the Pacific . Secretariat of the Pacific Regional Environment Programme. https://brb.sprep.org/content/use‐natural‐enemies‐manage‐widespread‐weeds‐pacific

[cobi14344-bib-0117] Secretariat of the Pacific Regional Environment Programme (SPREP) . (2021). Pacific Islands framework for nature conservation and protected areas 2021–2025. Secretariat of the Pacific Regional Environment Programme.

[cobi14344-bib-0143] Secretariat of the Pacific Regional Environment Programme (SPREP) . (2022). Create sustainable financing for invasive species management . https://brb.sprep.org/sites/default/files/2023‐09/Battlers‐Sustainable%20Digital%202022.pdf

[cobi14344-bib-0118] Steven, A. , Dyke, G. , Hardiman, L. , Hutchinson, D. , Kerblat, F. , Sims, N. , Smith, L. , & Zhu, J. (2019). Establishment of an Earth observation platform to support Pacific Island nations environmental, climate and livelihood needs—Consultation workshop—Final report . Commonwealth Scientific and Industrial Research Organisation. https://research.csiro.au/cceo/wp‐content/uploads/sites/252/2018/10/Final‐report‐EO4Pacific.pdf

[cobi14344-bib-0119] Takeda, S. (2010). Vegetation analysis and spatial modelling of threats of invasive species using geospatial technologies (GST): A case study of the Sigatoka sand dunes national park, Fiji Islands. University of the South Pacific. https://librarycat.usp.ac.fj/client/en_GB/search/asset/5199/0

[cobi14344-bib-0120] Tittensor, D. , Walpole, M. , Hill, S. , Boyce, D. , Britten, G. , Burgess, N. , Butchart, S. , Leadley, P. , Regan, E. , Alkemade, R. , Baumung, R. , Bellard, C. , Bouwman, A. , Bowles‐Newark, N. , Chenery, A. , Cheung, W. , Christensen, V. , Cooper, H. , Crowther, A. , & Ye, Y. (2014). A mid‐term analysis of progress toward international biodiversity targets. Science, 346, 241–244. 10.1126/science.1257484 25278504

[cobi14344-bib-0121] Tobin, P. C. (2018). Managing invasive species. F1000Research, 7, Article 1686. 10.12688/f1000research.15414.1 PMC6206619 30416712

[cobi14344-bib-0122] Tsai, F. , & Chou, M. J. (2006). Texture augmented analysis of high resolution satellite imagery in detecting invasive plant species. Journal of the Chinese Institute of Engineers, 29(4), 581–592.

[cobi14344-bib-0123] Turbelin, A. J. , Malamud, B. D. , & Francis, R. A. (2017). Mapping the global state of invasive alien species: Patterns of invasion and policy responses. Global Ecology and Biogeography, 26(1), 78–92.

[cobi14344-bib-0124] Turner, W. , Spector, S. , Gardiner, N. , Fladeland, M. , Sterling, E. , & Steininger, M. (2003). Remote sensing for biodiversity science and conservation. Trends in Ecology & Evolution, 18(6), 306–314.

[cobi14344-bib-0125] UN‐GGIM (United Nations Global Geospatial Information Management) . (2023). United Nations Integrated Geospatial Information Framework. Part 1: Overarching strategy—Second Edition 2023. United Nations.

[cobi14344-bib-0126] University of Newcastle . (2019). Pacific Research Road Map . https://www.newcastle.edu.au/__data/assets/pdf_file/0005/389111/Samoa‐Roadmap‐Low‐Res‐Single‐Pages.pdf

[cobi14344-bib-0127] US Forest Service . (2020). Pacific Islands Forest Health Highlights 2020 . https://www.fs.usda.gov/foresthealth/docs/fhh/PI_FHH_2020.pdf

[cobi14344-bib-0128] van Kleunen, M. , Dawson, W. , Essl, F. , Pergl, J. , Winter, M. , Weber, E. , Kreft, H. , Weigelt, P. , Kartesz, J. , Nishino, M. , Antonova, L. A. , Barcelona, J. F. , Cabezas, F. J. , Cárdenas, D. , Cárdenas‐Toro, J. , Castaño, N. , Chacón, E. , Chatelain, C. , Ebel, A. L. , … Pyšek, P. (2015). Global exchange and accumulation of non‐native plants. Nature, 525(7567), 100–103. 26287466 10.1038/nature14910

[cobi14344-bib-0129] Wallace, C. S. A. , Walker, J. J. , Skirvin, S. M. , Patrick‐Birdwell, C. , Weltzin, J. F. , & Raichle, H. (2016). Mapping presence and predicting phenological status of invasive buffelgrass in southern Arizona using MODIS, climate and citizen science observation data. Remote Sensing, 8(7), Article 524. 10.3390/rs8070524

[cobi14344-bib-0130] Weisberg, P. J. , Dilts, T. E. , Baughman, O. W. , Meyer, S. E. , Leger, E. A. , Van Gunst, K. J. , & Cleeves, L. (2017). Development of remote sensing indicators for mapping episodic die‐off of an invasive annual grass (*Bromus tectorum*) from the Landsat archive. Ecological Indicators, 79, 173–181.

[cobi14344-bib-0131] Weisberg, P. J. , Dilts, T. E. , Greenberg, J. A. , Johnson, K. N. , Pai, H. , Sladek, C. , Kratt, C. , Tyler, S. W. , & Ready, A. (2021). Phenology‐based classification of invasive annual grasses to the species level. Remote Sensing of Environment, 263, Article 112568.

[cobi14344-bib-0132] West, A. , Evangelista, P. , Jarnevich, C. , Kumar, S. , Swallow, A. , Luizza, M. , & Chignell, S. (2017). Using multi‐date satellite imagery to monitor invasive grass species distribution in post‐wildfire landscapes: An iterative, adaptable approach that employs open‐source data and software. International Journal of Applied Earth Observation and Geoinformation, 59, 135–146.

[cobi14344-bib-0133] Willmer, G. (2021). Train and retain: Forecasting disaster with geographic systems in the Pacific. Devex. https://www.devex.com/news/train‐and‐retain‐forecasting‐disaster‐with‐geographic‐systems‐in‐the‐pacific‐98876

[cobi14344-bib-0134] Worthy, K. , & Race, G. (2023). Building resilience in the Pacific. Geosaptial World. https://www.geospatialworld.net/prime/building‐resilience‐in‐pacific/

[cobi14344-bib-0135] Wu, Z. , Ni, M. , Hu, Z. , Wang, J. , Li, Q. , & Wu, G. (2019). Mapping invasive plant with UAV‐derived 3D mesh model in mountain area—A case study in Shenzhen Coast, China. International Journal of Applied Earth Observation and Geoinformation, 77, 129–139.

[cobi14344-bib-0136] Xu, T. , Wang, F. , Xie, L. , Yao, X. , Zheng, J. , Li, J. , & Chen, S. (2022). Integrating the textural and spectral information of UAV hyperspectral images for the improved estimation of rice aboveground biomass. Remote Sensing, 14(11), Article 2534.

[cobi14344-bib-0137] Zeng, Y. , Hao, D. , Huete, A. , Dechant, B. , Berry, J. , Chen, J. M. , Joiner, J. , Frankenberg, C. , Bond‐Lamberty, B. , Ryu, Y. , Xiao, J. , Asrar, G. R. , & Chen, M. (2022). Optical vegetation indices for monitoring terrestrial ecosystems globally. Nature Reviews Earth & Environment, 3(7), 477–493.

