# Supplementary material for: Capacity and capability of remote sensing to inform invasive plant species management in the Pacific Islands region
Source: Conserv Biol. 2024 Aug 21;39(1):e14344. doi: 10.1111/cobi.14344 (PMC11780207; doi:10.1111/cobi.14344)
Supplement: Supplementary file 1 — Supporting Information [file COBI-39-e14344-s001.docx]

**SUPPORTING INFORMATION**

**APPENDIX S1: Published Pacific-focused Literature on Invasive Plant Species**

Pacific-focused literature on invasive plant species that is available, including organizational reports released on request, and published academic theses related to invasive plant species.

We used Google Scholar, three regional virtual libraries, SPREP, SPC and USP, and the Landcare Research and Pacific GIS/RS Newsletter catalogue. Keywords were used in various combinations to identify relevant literature on invasive plant species (invasive plant species, invasive alien species, invasive species) and remote sensing (remote sensing, satellite imagery, earth observation, geospatial, GIS, mapping) techniques (phenology, machine learning, classification, structure, texture), relevant to invasive species management (management, mitigation, conservation) globally and across the Pacific Island region (Pacific, South Pacific, Oceania). Research articles, reports, and reviews on the topic of using remote sensing to map invasive plant species, were filtered from 2016 to 2022. The Landcare Research and Pacific GIS/RS Newsletters were searched manually, and additional literature and information was searched using cross-references of selected articles and reports. A total of 3651 results were obtained and filtered based on the appropriateness of the title, keywords and abstract. The papers that seemed most relevant to this reviews’ aims (477 papers) were then read in full and filtered to remove misaligned papers, leaving 116 papers in total.

From the 116, we organized a subset of literature, a total of 42 papers, that focused on the invasive plant species and their related management in the Pacific (Appendix S1, Table 1). This was based on the type of document, the topic (title, keywords and abstract), the study area and overall content. Table S1 provides the list of Pacific-based literature organized into themes. It is important to note these do not include policy frameworks and guidelines as this is later provided.

**Table S1. Subset of Pacific literature on invasive plant species.**

| **Title** | **Author(s), Year** | **Thematic Area** | **Location** |
| --- | --- | --- | --- |
| **Distribution Modelling** | | | |
| Will climate change impact the potential distribution of a native vine (*Merremia peltata*) which is behaving invasively in the Pacific region? | Taylor and Kumar (2016) | Species distribution modelling | Fiji, Hawaii, New Caledonia, Papua New Guinea, Solomon Islands and Vanuatu |
| Using range filling rather than prevalence of invasive plant species for management prioritisation: the case of *Spathodea campanulata* in Society Islands (South Pacific) | Pouteau, Meyer, and Larrue (2015) | Niche modelling, invasive species management | Tahiti, Moorea, and Raiatea (Society Islands) |
| **Species Biology, Behavior and Distribution** | | | |
| Seed Rain, Dispersal Distance, and Germination of the Invasive Tree *Spathodea campanulata* on the Island of Tahiti, French Polynesia (South Pacific) | Sébastien et al. (2021) | Species review, species behavior | French Polynesia |
| Invasive alien palm Pinanga coronata threatens native tree ferns in an oceanic island rainforest. | M. J. B. Dyer, Keppel, Tuiwawa, Vido, and Boehmer (2018) | Biodiversity loss, management | Fiji |
| Abiotic effects of the invasive alien palm *Pinanga coronata* in the Colo-i-Suva Forest Reserve, Fiji. [Academic Research] | Gopaul (2017) | Abiotic analysis, invasive species management | Fiji |
| The distribution of the invasive palm Pinanga coronata and its effects on native tree ferns in the Colo-i-Suva area, Viti Levu, Fiji. [Academic Thesis] | M. J. Dyer (2016) | Biodiversity loss, forest management | Fiji |
| Elevational distribution and photosynthetic characteristics of the invasive tree Spathodea campanulata on the island of Tahiti (South Pacific Ocean) | Larrue, Daehler, Meyer, Pouteau, and Voldoire (2016) | Invasive species, elevation ranges, micro-climate | Tahiti |
| Biology and Impacts of Pacific Island Invasive Species. 13. *Mikania micrantha Kunth* (Asteraceae) | Day et al. (2016) | Species review | Pacific Island region |
| Ticking time bombs – current and potential future impacts for four invasive plant species on the biodiversity of lowland tropical rainforests in south-east Viti Levu, Fiji | Keppel and Walting (2011) | Invasive species status, invasive species management | Fiji |
| Pacific Island Forests: Successionally Impoverished and Now Threatened to be Overgrown by Aliens? | Mueller-Dombois (2008) | Island biogeography model, alien plant invasion, island vegetation | Pacific Island region |
| Valuing Invasives: Understanding the *Merremia peltata* Invasion in Post-Colonial Samoa [Academic Research] | Kirkham (2004) | Species review, island vegetation | Samoa |
| African Tulip Tree in the Fijian Islands | Auld and Nagatalevu-Seniloli (2003) | Invasive species, weed management, distribution | Fiji |
| **Invasive Species Management** | | | |
| Pacific Island Perspectives on Invasive Species and Climate Change [Book Chapter] in Social and Ecological Interactions in the Galapagos Islands | Brewington et al. (2023) | Pacific Island resilience, climate-literate resource management | Guam, American Samoa, Commonwealth State of the Northern Mariana Islands, Palau, Federated States of Micronesia and Marshall Islands (US-Affiliated Pacific Islands) |
| Where to invade next: Inaction on biological invasions threatens sustainability in a small island developing state of the tropical South Pacific [Book Chapter] in Sustainable Development: Asia-Pacific Perspectives | Lenz et al. (2022) | Sustainable development, biodiversity conservation | Pacific Island Countries and Fiji |
| Pacific Islands Forest Health Highlights 2020 | US Forest Service (2020) | Invasive species management, biocontrol, forest resources | American Samoa, Commonwealth State of the Northern Mariana Islands, Palau, Federated Sates of Micronesia and Marshall Islands (US-Affiliated Pacific Islands) |
| State of environment and conservation in the Pacific Islands: 2020 Regional Report | SPREP (2020a) | Biodiversity, conservation management, biocontrol | SPREP member countries |
| Battling invasive species in the Pacific | Moverley (2019) | Management | Pacific Island Countries |
| Impact and Management of Invasive Alien Plants in Pacific Island Communities | Orapa (2017) | Management | SPREP member countries |
| Restoration Plan Consultation Report for the OLPP NP | Atherton (2015) | Management, engagement, restoration | Samoa |
| Invading the Pacific: biological and cultural dimensions of invasive species in the Pacific region | Russell (2004) | Management, social dimension, cultural dimension | Pacific Island Countries |
| Preliminary review of the invasive plants in the Pacific Islands (SPREP Member Countries) | Meyer (2000) | Management, review | SPREP member countries |
| **Invasive Species Management (Cost-benefit analysis, livelihood assessments, taxonomic and functional analysis, etc.)** | | | |
| A functional trait-based approach to assess the impact of an alien palm invasion on plant and soil communities on a South Pacific Island | Forey, Lodhar, Gopaul, Boehmer, and Chauvat (2021) | above-belowground interactions, biological invasion, functional traits, plant communities | Fiji |
| Using expert knowledge and field surveys to guide management of an invasive alien palm in a pacific Island lowland rainforest in Island invasives: Scaling up to meet the challenge | M. Dyer, Keppel, Tuiwawa, Vido, and Boehmer (2019) | Biodiversity loss, invasive species management | Fiji |
| Population structure, allometry, and spread of alien ivory cane palm, *Pinanga coronata*, in a protected forest landscape on Viti Levu, Fiji. [Academic Thesis] | Hanson (2017) | Population structure, invasive species management | Fiji |
| Risk assessment of the invasive alien ivory-cane Palm (*Pinanga coronata*) in the islands of the tropical South Pacific. [Academic Thesis] | Lenz (2016) | Risk assessment, invasive species management | Fiji |
| Cost-benefit analysis of managing the invasive African tulip tree (*Spathodea campanulata*) in the Pacific | Brown and Daigneault (2014) | Cost-benefit analysis, Invasive species management | Fiji |
| Invasions and impacts of exotic plants in the Pacific Islands [Conference Proceedings] | Denslow (2008) | Management, restoration, diversity | Pacific Island Countries including Hawaii |
| A risk assessment system for screening out invasive pest plants from Hawaii and other Pacific Island countries | Daehler, Denslow, Ansari, and Kuo (2004) | Management, risk-assessment | Pacific Island Countries including Hawaii |
| **Invasive Species Management (Biocontrol)** | | | |
| Feasibility of biological control of taro vine, *Epipremnum pinnatum (L.) Engl. Cv. Aureum* in the Pacific region [Landcare Research Contract Report] | McGrannachan, Mitchell, and Probst (2021) | Species review, biological control | Pacific Island countries |
| Use natural enemies to manage widespread weeds in the Pacific | SPREP (2020b) | Invasive species management, biological control | SPREP member countries |
| West African arthropods hold promise as biological control agents for an invasive  tree in the Pacific Islands | Paterson et al. (2017) | Biological control, African tulip | Pacific Island countries |
| Biological control of weeds in the 22 Pacific Island countries and territories: current status and future prospects | Day and Winston (2016) | Invasive species management, biocontrol | Pacific Island countries |
| Prioritisation of targets for biocontrol of weeds on Pacific Islands [Landcare Research Contract Report] | Quentin Paynter (2010) | Biocontrol, invasive species management | Pacific Island countries |
| Prospects for biological control of Merremia peltata [Landcare Research Contract Report] | Q. Paynter, Harman, and Waipara (2006) | Species review, biological control | Pacific Island countries |
| The need to build biological control capacity in the Pacific [Conference Proceedings] | Dovey et al. (2004) | Biological control, capacity, biocontrol biodiversity | Pacific Islands countries |
| **Invasive Species Management (GIS Analysis and Remote Sensing)** | | | |
| Assessing the impacts of invasive alien tree species in lowland forest ecosystem in order to improve restoration strategies in Samoa. [Academic Thesis] | Pisi (2019) | Management, statistical analysis, remote sensing | Samoa |
| The threat by the invasive African tulip tree, *Spathodea campanulata P.Beauv*., for the critically endangered Fijian tree, *Ptercymbium oceanicum A. C. Sm.*; revisiting an assessment based on expert knowledge after extensive field surveys | Keppel, Peters, Taoi, Raituku, and Thomas-Moko (2021) | Conservation assessment, GIS, field survey | Fiji |
| Vegetation Analysis and Spatial Modelling of Threats of Invasive Species Using Geospatial Technologies (GST): A Case Study of the Sigatoka Sand Dunes National Park, Fiji Islands | Takeda (2010) | Conservation management, satellite imagery, GIS | Fiji |
| Mapping Merremia Peltata in Ole Pupu Pue National Park: Recommendations for Ecological Restoration. Sustainable Forest Management Component: Part 1 Final Report [Forest Preservation Programme FY2010 Report] | Asia Air Survey Co Ltd. (2014) | Remote sensing, satellite imagery, management | Samoa |
| Novel ecosystems in the Pacific Islands: Assessing loss, Fragmentation and Alteration of Native Forests by Invasive Alien Plants on the Island of Moorea (French Polynesia) [Book Chapter] in  Biodiversity and Societies in the Pacific Islands | Pouteau, Meyer, Fourdrigniez, and Taputuarai (2013) | Remote sensing, satellite imagery, landscape management | French Polynesia |
| The importance of novel and hybrid habitats for plant conservation on islands: a case study from Moorea (South Pacific) | Meyer, Pouteau, Spotswood, Taputuarai, and Fourdrigniez (2015) | Anthropogenic disturbances, habitat mapping, naturalized flora, plant invasion | French Polynesia |
| Spatial patterns of presence, abundance, and richness of invasive woody plants in relation to urbanization in a tropical island setting. | Lowry et al. (2020) | Invasive species status, Urban-rural spatial pattern, GIS | Fiji |

**REFERENCE LIST**

Abdi, H., & Williams, L. J. (2010). Principal component analysis. *Wiley interdisciplinary reviews: computational statistics, 2*(4), 433-459.

Abdullah, H., Skidmore, A. K., Darvishzadeh, R., & Heurich, M. (2019). Sentinel-2 accurately maps green-attack stage of European spruce bark beetle (Ips typographus, L.) compared with Landsat-8. *Remote Sensing in Ecology and Conservation, 5*(1), 87-106. doi:<https://doi.org/10.1002/rse2.93>

Asia Air Survey Co Ltd. (2014). *Mapping Merremia Peltata in Ole Pupu Pue National Park: Recommendations for Ecological Restoration*. Retrieved from Samoa:

Atherton, J. (2015). *Restoration Plan Consultation Report for the OLPP NP*. Retrieved from Apia, Samoa: <https://brb.sprep.org/sites/default/files/2021-12/restoration-plan-consultation-report-pupu-pue-national-park.pdf>

Atkinson, P. M., & Tatnall, A. R. L. (1997). Introduction Neural networks in remote sensing. *International Journal of Remote Sensing, 18*(4), 699-709. doi:10.1080/014311697218700

Auld, B., & Nagatalevu-Seniloli, M. (2003). African Tulip Tree in the Fijian Islands. In M. Labrada (Ed.), *Weed Management for Developing Countries, Addendum I* (pp. 63-65): FAO.

Breiman, L. (2001). Random Forests. *Machine Learning, 45*(1), 5-32. doi:10.1023/A:1010933404324

Brewington, L., Eichelberger, B., Read, N., Parsons, E., Kerkering, H., Martin, C., . . . Burgett, J. (2023). Pacific Island Perspectives on Invasive Species and Climate Change. In S. J. Walsh, C. F. Mena, J. R. Stewart, & J. P. Muñoz Pérez (Eds.), *Island Ecosystems: Challenges to Sustainability* (pp. 59-78). Cham: Springer International Publishing.

Brown, P., & Daigneault, A. (2014). Cost-benefit analysis of managing the invasive African tulip tree (Spathodea campanulata) in the Pacific. *Environmental Science & Policy*(39), 65-76. doi:<http://dx.doi.org/10.1016/j.envsci2014.02.004>

Cheng, Q., Varshney, P. K., & Arora, M. (2006). Logistic Regression for Feature Selection and Soft Classification of Remote Sensing Data. *Geoscience and Remote Sensing Letters, IEEE, 3*, 491-494. doi:10.1109/LGRS.2006.877949

Chetty, S., Mutanga, O., & Lottering, R. (2021). Detecting and mapping invasive Parthenium hysterophorus L. along the northern coastal belt of KwaZulu-Natal, South Africa using image texture. *Scientific African, 13*, e00966. doi:<https://doi.org/10.1016/j.sciaf.2021.e00966>

Daehler, C. C., Denslow, J. S., Ansari, S., & Kuo, H.-C. (2004). A Risk-Assessment System for Screening out Invasive Pest Plants from Hawaii and Other Pacific Islands. *Conservation Biology, 18*(2), 360-368. Retrieved from <http://www.jstor.org.ezproxy.newcastle.edu.au/stable/3589213>

Dahal, D., Pastick, N. J., Boyte, S. P., Parajuli, S., Oimoen, M. J., & Megard, L. J. (2022). Multi-Species Inference of Exotic Annual and Native Perennial Grasses in Rangelands of the Western United States Using Harmonized Landsat and Sentinel-2 Data. *Remote Sensing, 14*(4), 807. Retrieved from <https://www.mdpi.com/2072-4292/14/4/807>

Day, M. D., Clements, D. R., Gile, C., Senaratne, W. K. A. D., Shen, S., Weston, L. A., & Zhang, F. (2016). Biology and Impacts of Pacific Islands Invasive Species. 13. <i>Mikania micrantha</i> Kunth (Asteraceae). *Pacific Science, 70*(3), 257-285, 229. Retrieved from <https://doi.org/10.2984/70.3.1>

Day, M. D., & Winston, R. L. (2016). Biological control of weeds in the 22 Pacific island countries and territories: current status and future prospects. *NeoBiota, 30*, 167-192. Retrieved from <https://doi.org/10.3897/neobiota.30.7113>

Denslow, J. S. (2008). *Invasions and impacts of exotic plants in the Pacific islands.* Paper presented at the Proceedings of the 16th Australian Weeds Conference, Brisbane.

Dovey, L., Orapa, W., Randall, S., Cullen, J. M., Briese, D. T., Kriticos, D. J., . . . Scott, J. K. (2004). *The need to build biological control capacity in the Pacific*.

Duncan, P., Podest, E., Esler, K. J., Geerts, S., & Lyons, C. (2023). Mapping Invasive Herbaceous Plant Species with Sentinel-2 Satellite Imagery: Echium plantagineum in a Mediterranean Shrubland as a Case Study. *Geomatics, 3*(2), 328-344. Retrieved from <https://www.mdpi.com/2673-7418/3/2/18>

Dyer, M., Keppel, G., Tuiwawa, M., Vido, S., & Boehmer, H. J. (2019). Using expert knowledge and field surveys to guide management of an invasive alien palm in a Pacific Island lowland rainforest. In CR Veitch, MN Clout, AR Martin, J. Russell, & C. West (Eds.), *Island invasives: scaling up to meet the challenge. Proceedings of the international conference on island invasives* (pp. 417-422). Gland, Switzerland.: IUCN.

Dyer, M. J. (2016). *The distribution of the invasive palm Pinanga coronata and its effects on native tree ferns in the Colo-i-Suva area, Viti Levu, Fiji.* (Honours). The University of the South Pacific, Faculty of Science, Technology, Australia.

Dyer, M. J. B., Keppel, G., Tuiwawa, M., Vido, S., & Boehmer, H. J. (2018). Invasive alien palm *Pinanga coronata* threatens native tree ferns in an oceanic island rainforest. *Australian Journal of Botany, 66*(8), 647-656. doi:<https://doi.org/10.1071/BT18088>

Forey, E., Lodhar, S., Gopaul, S., Boehmer, H. J., & Chauvat, M. (2021). A functional trait-based approach to assess the impact of an alien palm invasion on plant and soil communities on a South Pacific island. *Austral Ecology, 46*(3), 398-410. doi:<https://doi.org/10.1111/aec.12995>

Gandhi, R. (2018). Support Vector Machine - Introductions to Machine Learning Algorithms. Retrieved from <https://towardsdatascience.com/support-vector-machine-introduction-to-machine-learning-algorithms-934a444fca47>

Gopaul, S. (2017). *Abiotic effects of the invasive alien palm (Pinanga coronata) in the Colo-i-suva forest reserve, Fiji.* (Master of Science). The University of the South Pacific, Suva, Fiji. Retrieved from <http://uspaquatic.library.usp.ac.fj/gsdl/collect/usplibr1/index/assoc/HASHe8dc.dir/doc.pdf>

Hanson, G. C. (2017). *Population structure, allometry, and spread of alien ivory cane palm, Pinanga coronata, in a protected forest landscape on Viti Levu, Fiji.* (Master in Environmental Science). The University of the South Pacific, Fiji. Retrieved from <http://www.pimrisregional.library.usp.ac.fj/gsdl/collect/usplibr1/index/assoc/HASH0194.dir/doc.pdf>

Harrison, O. (2018). Machine Learning Basics with the K-Nearest Neighbors Algorithm. Retrieved from <https://towardsdatascience.com/machine-learning-basics-with-the-k-nearest-neighbors-algorithm-6a6e71d01761>

Huang, Q., Peng, L., Huang, K., Deng, W., & Liu, Y. (2022). Generalized Additive Model Reveals Nonlinear Trade-Offs/Synergies between Relationships of Ecosystem Services for Mountainous Areas of Southwest China. *Remote Sensing, 14*(12). doi:10.3390/rs14122733

Huete, A. R. (1988). A soil-adjusted vegetation index (SAVI). *Remote Sensing of Environment, 25*(3), 295-309. doi:<https://doi.org/10.1016/0034-4257(88)90106-X>

Hunt, Jr., Hively, W., Fujikawa, S., Linden, D., Daughtry, C., & McCarty, G. (2010). Acquisition of NIR-Green-Blue Digital Photographs from Unmanned Aircraft for Crop Monitoring. *Remote Sensing, 2*. doi:10.3390/rs2010290

Jin, S., & Sader, S. (2005). Comparison of time series Tasseled Cap wetness and the normalized difference moisture index in detecting forest disturbances. *Remote Sensing of Environment, 94*, 364-372. doi:10.1016/j.rse.2004.10.012

Kattenborn, T., Eichel, J., Wiser, S., Burrows, L., Fassnacht, F. E., & Schmidtlein, S. (2020). Convolutional Neural Networks accurately predict cover fractions of plant species and communities in Unmanned Aerial Vehicle imagery. *Remote Sensing in Ecology and Conservation, 6*(4), 472-486. doi:<https://doi.org/10.1002/rse2.146>

Keppel, G., Peters, S., Taoi, J., Raituku, N., & Thomas-Moko, N. (2021). The threat by the invasive African tulip tree, Spathodea campanulata P.Beauv., for the critically endangered Fijian tree, Pterocymbium oceanicum A.C.Sm.; revisiting an assessment based on expert knowledge after extensive field surveys. *Pacific Conservation Biology*. Retrieved from <https://doi.org/10.1071/PC20068>

Keppel, G., & Walting, D. (2011). Ticking time bombs - current and potential future impacts of four invasive plant species on the biodiversity of lowland tropical rainforests in south-east Viti Levu, Fiji. *The South Pacific Journal of Natural and Applied Sciences, 29*, 43-45. doi:<http://10.1071/SP11009>

Kirkham, W. S. (2004). Situating the Merremia Peltata Invasion in Samoa. *Geographical Review, 94*(2), 218-228. doi:10.1111/j.1931-0846.2004.tb00168.x

Kupidura, P. (2019). The Comparison of Different Methods of Texture Analysis for Their Efficacy for Land Use Classification in Satellite Imagery. *Remote Sensing, 11*(10). doi:10.3390/rs11101233

Larrue, S., Daehler, C., Meyer, J.-Y., Pouteau, R., & Voldoire, O. (2016). Elevational distribution and photosynthetic characteristics of the invasive tree Spathodea campanulata on the island of Tahiti (South Pacific Ocean). *NeoBiota*(30), 127-149. doi:10.3897/neobiota.30.8201

Lenz, M. I. (2016). *Risk assessment of the invasive alien ivory-cane Palm (Pinanga coronata) in the islands of the tropical South Pacific. .* (BA Geograhy). The University of the South Pacific,

Georg-August University of Goettingen, Germany. , Goettingen, Germany.

Lenz, M. I., Galvin, S., Keppel, G., Gopaul, S., Kowasch, M., Dyer, M. J., . . . Boehmer, H. J. (2022). Where to invade next: Inaction on biological invasions threatens sustainability in a small island developing state of the tropical South Pacific. In P. S. Low (Ed.), *Sustainable Development: Asia-Pacific Perspectives* (pp. 393-406). Cambridge: Cambridge University Press.

Liu, Y., Gong, W., Hu, X., & Gong, J. (2018). Forest Type Identification with Random Forest Using Sentinel-1A, Sentinel-2A, Multi-Temporal Landsat-8 and DEM Data. *Remote Sensing, 10*(6). doi:10.3390/rs10060946

Lottering, R. T., Govender, M., Peerbhay, K., & Lottering, S. (2020). Comparing partial least squares (PLS) discriminant analysis and sparse PLS discriminant analysis in detecting and mapping Solanum mauritianum in commercial forest plantations using image texture. *ISPRS Journal of Photogrammetry and Remote Sensing, 159*, 271-280. doi:<https://doi.org/10.1016/j.isprsjprs.2019.11.019>

Lowry, B. J., Lowry, J. H., Jarvis, K. J., Keppel, G., Thaman, R. R., & Boehmer, H. J. (2020). Spatial patterns of presence, abundance, and richness of invasive woody plants in relation to urbanization in a tropical island setting. *Urban Forestry & Urban Greening, 48*, 126516. doi:<https://doi.org/10.1016/j.ufug.2019.126516>

Marzialetti, F., Frate, L., De Simone, W., Frattaroli, A. R., Acosta, A. T., & Carranza, M. L. (2021). Unmanned Aerial Vehicle (UAV)-Based Mapping of Acacia saligna Invasion in the Mediterranean Coast. *Remote Sensing, 13*(17). doi:10.3390/rs13173361

Matsushita, B., Yang, W., Chen, J., Onda, Y., & Qiu, G. (2007). Sensitivity of the enhanced vegetation index (EVI) and normalized difference vegetation index (NDVI) to topographic effects: a case study in high-density cypress forest. *Sensors, 7*(11), 2636-2651.

McGrannachan, C., Mitchell, C., & Probst, C. (2021). *Feasibility of biological control of taro vine, Epipremnum pinnatum (L.) Engl. cv. Aureum in the Pacific region*. Retrieved from

McIntyre, D. (2015). *Application of high resolution remote sensing to detect and map the pasture weed Paterson’s curse (Echium plantagineum) in Western Australia*.

Meyer, J.-Y. (2000). Preliminary review of the invasive plants in the Pacific Islands (SPREP Member Countries). In G. Sherley (Ed.), *Invasive Species in the Pacific. A technical review and draft regional strategy*. Apia: South Pacific Regional Environmental Program.

Meyer, J.-Y., Pouteau, R., Spotswood, E., Taputuarai, R., & Fourdrigniez, M. (2015). The importance of novel and hybrid habitats for plant conservation on islands: a case study from Moorea (South Pacific). *Biodiversity and Conservation, 24*(1), 83-101. doi:10.1007/s10531-014-0791-6

Moody, J. (2019). What does RMSE really mean? Retrieved from <https://towardsdatascience.com/what-does-rmse-really-mean-806b65f2e48e>

Moverley, D. (2019). Battling invasive species in the Pacific. In C. R. Veitch, M. N. Clout, A. R. Martin, J. C. Russell, & C. J. West (Eds.), *Island invasives: scaling up to meet the challenge* (Vol. 62, pp. 658-662). Gland, Switzerland: IUCN.

Mueller-Dombois, D. (2008). Pacific Island Forests: Successionally Impoverished and Now Threatened to Be Overgrown by Aliens? *Pacific Science*, 303-308. doi:10.2984/1534-6188(2008)62[303:PIFSIA]2.0.CO;2

Orapa, W. (2017). Impact and Management of Invasive Alien Plants in Pacific Island Communities. In C. A. S. Ellison, K. V; Murphy, S. T. (Ed.), *Invasive Alien Plants*: CAB International.

Paterson, I. D., Paynter, Q., Neser, S., Akpabey, F. J., Orapa, W., & Compton, S. G. (2017). West African Arthropods Hold Promise as Biological Control Agents for an Invasive Tree in the Pacific Islands. *African Entomology, 25*(1), 244-247, 244. Retrieved from <https://doi.org/10.4001/003.025.0244>

Paynter, Q. (2010). *Priotisation of targets for biological controls of weeds on Pacific Islands.* Retrieved from Auckland, New Zealand: <https://www.pacificinvasivesinitiative.org/site/pii/files/resources/publications/other/Prioritisation%20of%20targets%20for%20biological%20control%20of%20weeds%20on%20Pacific%20Islands.pdf>

Paynter, Q., Harman, H., & Waipara, N. (2006). *Prospects for biological control of Merremia peltata*. Retrieved from Auckland, New Zealand: <http://www.botany.hawaii.edu/basch/uhnpscesu/pdfs/sam/Paynter2006AS.pdf>

Peerbhay, K., Germishuizen, I., Lottering, R., & Naicker, R. (2022). Remote sensing wattle rust induced defoliation across black wattle timber plantations in Southern Africa. *International Journal of Remote Sensing, 43*(6), 2212-2226. doi:10.1080/01431161.2022.2058891

Pisi, J. T. (2019). *Assessing the impacts of invasive alien tree speices in lowland forest ecosystem in order to improve restoration strategies in Samoa.* (Global Environmental Management). Hokkaido University, Sapporo, Hokkaido.

Pouteau, R., Meyer, J.-Y., Fourdrigniez, M., & Taputuarai, R. (Eds.). (2013). *Novel ecosystems in the Pacific Islands: assessing loss, fragmentation and alteration of native forests by invasive alien plants on the island of Moorea (French Polynesia)*: Presses Universitaires de Provence & The Australian National University e-Press.

Pouteau, R., Meyer, J.-Y., & Larrue, S. (2015). Using range filling rather than prevalence of invasive plant species for management prioritisation: the case of Spathodea campanulata in the Society Islands (South Pacific). *Ecological Indicators, 54*, 87-95. doi:<https://doi.org/10.1016/j.ecolind.2015.02.017>

Russell, J. C. (2004). Invading the Pacific: biological and cultural dimensions of invasive species in the Pacific Region. *Graduate Journal of Asia-Pacific Studies, 2*(2), 77-94.

Saba, F., Latifi, H., Valadan Zoej, M. J., & Esmaili, R. (2022). Mapping dead understorey Buxus hyrcana Pojark using Sentinel-2 and Sentinel-1 data. *Forestry: An International Journal of Forest Research, 96*(2), 228-248. doi:10.1093/forestry/cpac049

Sébastien, L., Jean-Yves, M., Boris, F., Curtis, D., Julien, C., Maurille, F., & Lucas, M. (2021). Seed Rain, Dispersal Distance, and Germination of the Invasive Tree *Spathodea campanulata* on the Island of Tahiti, French Polynesia (South Pacific). *Pacific Science, 74*(4), 405-417. doi:10.2984/74.4.8

SPREP. (2020a). *State of environment and conservation in the Pacific Islands: 2020 Regional Report*. Retrieved from Apia, Samoa: <https://library.sprep.org/sites/default/files/2021-03/SOE-conservation-pacific-regional-report.pdf>

SPREP. (2020b). *Use natural enemies to manage widespread weeds in the Pacific*. Retrieved from Apia: <https://brb.sprep.org/content/use-natural-enemies-manage-widespread-weeds-pacific>

Takeda, S. (2010). *Vegetation analysis and spatial modelling of threats of invasive species using geospatial technologies (GST): A case study of the Sigatoka sand dunes national park, Fiji Islands.* (Doctor of Philosophy in Geography). University of the South Pacific, Suva, Fiji.

Taylor, S., & Kumar, L. (2016). Will climate change impact the potential distribution of a native vine (Merremia peltata) which is behaving invasively in the Pacific region? *Ecology and Evolution, 6*(3), 742-754. doi:<https://doi.org/10.1002/ece3.1915>

US Forest Service. (2020). *Pacific Islands Forest Health Highlights 2020*. Retrieved from

Wu, Z., Ni, M., Hu, Z., Wang, J., Li, Q., & Wu, G. (2019). Mapping invasive plant with UAV-derived 3D mesh model in mountain area—A case study in Shenzhen Coast, China. *International Journal of Applied Earth Observation and Geoinformation, 77*, 129-139. doi:<https://doi.org/10.1016/j.jag.2018.12.001>

**APPENDIX S2: AVAILABLE GEOSPATIAL DATA AND RELEVANT CATALOGUES FOR THE PACIFIC ISLANDS REGION**

| Dataset Name | Detail | Resolution | Availability | Source |
| --- | --- | --- | --- | --- |
| SATELLITE IMAGERY | | | | |
| Landsat 7 Enhanced Thematic Mapper Plus (ETM+) | Launched 1999 carrying ETM+ sensor with 8 spectral bands. | 30m (multispectral)  15m (panchromatic)  16-day revisit  8-bit radiometric resolution | Global coverage, publicly available | NASA/USGS |
| Landsat 8 Operational Land Imager (OLI) and Thermal Infrared Sensor (TIRS) | Launched 2013, carrying two sensors that product 11 spectral bands. OLI measures visible, near infrared and shortwave infrared portions (VNIR, NIR, SWIR) of the spectrum. TRIS measures land surface temperature through two thermal bands. | 30m (multispectral)  15m (panchromatic)  16-day revisit  12-bit radiometric resolution  185km swath width | Global coverage, publicly available | NASA/USGS |
| Landsat 9 (OLI2-2) and (TIRS-2) | Launched 2021, instruments onboard are improved replicas (OLI-2, TIRS-2) of Landsat 8 system and carries 11 spectral bands. | 30m (multispectral)  15m (panchromatic)  16-day revisit  14-bit radiometric resolution  185km swath width | Global coverage, publicly available | NASA/USGS |
| Copernicus Sentinel-2 | Launched in 2015 comprises of a constellation of two polar orbiting satellites (2A and 2B) imaging land and coastal areas in sun-synchronous orbit and produces 13 spectral bands | 10m, 20m, 60m  5-day revisit  12-bit radiometric resolution  290km swath width | Global coverage, publicly available | European Space Agency |
| JAXA ALOS-2 PALSAR-2 | The Advanced Land Observing Satellite-2 (ALOS-2) is a follow-up missing from ALOS, contributing to cartography, regional observation, disaster monitoring and resource surveys. ALOS-2 was launched in 2014 with the L-band Synthetic Aperture Radar (PALSAR-2), capable of night and day observation across all weather conditions. | 3m, 6m, 10m, 60m, 100m resolution  14-day revisit  30km, 40km, 50km, 350km, 490km swath width | Global coverage, publicly available | Japanese Aerospace Exploration Agency (JAXA) |
| EO-1 Hyperion | Launched in 2003, Hyperion collects 220 unique spectral channels ranging from 0.357 to 2.576mn with a 10-nm bandwidth. The instrument operates in a push broom fashion. | 30m spatial resolution  7.7km x 42km scene size | Global coverage available (2003-2011), publicly available | NASA/USGS |
| MAXAR  WorldView-1, 2,3 | The WorldView constellation produces commercial high-resolution satellite imagery for environment-monitoring from Maxar in the United States. WorldView-1 launched in 2007 and is a panchromatic-only instrument still in operation. WorldView-2 and WorldView-3 launched in 2009 and 2014, respectively. | 8 spectral bands  0.46 – 0.50m (panchromatic)  1.8m (multispectral)  16.4-17.6kmkm swath width  1-1.7-day revisit  11-bit | Regional coverage available for purchase, PICT’s coverage limited to area/year | MAXAR (purchase), Local Ministries (Lands Department), SPC, SPREP, USP |
| Moderate Resolution Imaging Spectroradiometer (MODIS) | Two spaceflight units above the Terra and Aqua satellite have been launched for the purpose of atmospheric, land and ocean imaging in a single instrument. The instrument provides high radiometric sensitivity (36 spectral bands) | 36 spectral bands  250 m (band 1-2)  500m (band 3-7)  1km (band 8-36)  2330 km swath  1–2-day revisit  12-bit | Global coverage available, publicly available | NASA/USGS |
| IKONOS | High-resolution satellite that is decommissioned, previously operated by MAXAR. The satellite can yield data for nearly all aspects of environmental study. | 3.2m (multispectral, NIR)  0.82m (panchromatic)  3-day revisit | Regional coverage available for purchase, PICT’s coverage limited to area/year | MAXAR (purchase), Local Ministry,  SPC |
| Quickbird | High-resolution commercial sensor with 5 bands operated by MAXAR launched in 2002 and has since been decommissioned in 2015. | 1-3.5 days revisit (depending on latitude)  0.65m (panchromatic)  2.62m (multispectral)  11-bit | Regional coverage available for purchase, PICT’s coverage limited to area/year | MAXAR (purchase), Local Ministry, SPC |
| Global Ecosystem Dynamics Investigation (GEDI) | Space-borne high resolution lidar observation of the 3D structure of the Earth. Measures forest canopy height, canopy vertical structure, and surface elevation. | 25m spot footprint | Globally available, publicly available |  |
| LiDAR Series | Contracted parties. | Specific to contract sensor resolution | Samoa (Government)  Niue Government  Tonga (Government, SPC)  Marshall Islands (Government, SPC)  Fiji (not national coverage) | Local Ministry.  SPC |
| GEOSPATIAL ENVIRONMENTAL DATA CATALOGUES | | | | |
| [WorldClim](https://www.worldclim.org/) | Database of high spatial resolution global weather, climate, and elevation data. Contains both historical (1970-2000) and future (CMIP 6 downscaled) conditions. | 30 seconds (~1km)  2.5 minutes (~4.5km)  5 minutes (~9km)  10 minutes (~18.5km) | Global coverage, publicly available |  |
| Soil Geographic Database (ISRIC) | In collaboration with partners, ISRIC has compiled and harmonized global data on soils and their properties at local area, country, and global level. | Variety of formats  250m – 5km | Global coverage, publicly available. | [ISRIC](https://data.isric.org/geonetwork/srv/eng/catalog.search#/home) |
| ESRI 10m Landcover (2020) | A global 10m landcover classification map that has been generated using AI to Sentinel-2 2020 scene collection on Microsoft Planetary Computer. The output provides a 10-class map of the surface, including vegetation type, bare surface, crop and urban areas. | 10m resolution (Sentinel-2)  10 unique landcover classes | Global coverage for 2020, publicly available | ESRI |
| REGIONAL DATA CATAGLOGUES | | | | |
| SPREP Pacific Environment Data Portal (INFORM) | Connects 14 national environment portals and provides tools for monitoring, evaluation, and analysis. Regional and national datasets available based on project activities and priorities. | Variety of formats  Spatial: Vector, Raster  Non-spatial: CSV, PDF, DOCX | Regional, national data availability | SPREP [INFORM](https://pacific-data.sprep.org/search?f%5B0%5D=content_type%3Adataset) |
| Ridge to Reef State of the Coast | A regional database that houses a variety of environmental datasets with few species-specific data. These datasets were not collected for the purpose of SDM or species detection. This portal is currently not operational. | Variety of formats  Spatial: Vector, Raster  Non-spatial: CSV, PDF, DOCX | Selected countries (project dependent) | [State of the Coast](http://r2r.spc.int/) |
| Global Biodiversity Information Facility (GBIF) | An international network and data infrastructure funded by the world's governments and aimed at providing anyone, anywhere, open access to data about all types of life on Earth. | Vector dataset | Global coverage, publicly available | GBIF |
| [Pacific Biodiversity Information Pacific (PBIF)](https://pbif.sprep.org/) | Presents all Pacific biodiversity data that is available on GBIF. | Vector dataset | Regional coverage, publicly available | PGBIF |
| Pacific Data Hub (PDH) | Central repository of data about the Pacific from the Pacific. Contains data and information from key areas including population statistics, fisheries, climate change, disaster risk, conservation of plant genetic resources for food and human rights. | Variety of formats Spatial: Vector, Raster, GEOJSON  Non-spatial: CSV, PDF, DOCX, XLS | Regional coverage, national coverage, publicly available | [SPC Pacific Data Hub](https://pacificdata.org/) |

**APPENDIX S3: RELEVANT POLICIES, STRATEGIES AND FRAMEWORKS FOR INVASIVE PLANT SPECIES**

**Table S3.A: National Biodiversity Strategy and Action Plan (NBSAP), National Invasive Species Action Plans and Linked Frameworks**

| **Plan** | **Pacific Island Country (Latest Submission Year)** | **Invasive Species as a Priority in Plan** | **National Invasive Species Action Plan (Year)** | **Linked Domestic Frameworks/Strategies/Policies to NBSAP** |
| --- | --- | --- | --- | --- |
| National Biodiversity Strategy and Action Plans | Fiji (2020-2025) | X |  | Fiji Green Growth Framework  Fiji Low Emission Development Strategy 2018-2050  Fiji National Adaptation Plan Framework (2017)  Fiji 5-Year National Development Plan (2017-2021)  Fiji 20-Year National Development Plan (2017-2036)  Endangered and Protected Species Act 2002  Endangered and Protected Species Regulations 2003  Forest Policy (draft) 2007 |
|  | Cook Islands (2016) | X | [2019-2025](https://chm.cbd.int/api/v2013/documents/B63E7679-3021-D4C6-9E51-67575CF0AB8E/attachments/213276/Cook%20Is%20NISSAP%202019%20-%2005.pdf) | Offshore Fisheries Policy  Te Kaveinga Nui – National Sustainable Development Plan  Traditional Knowledge Act (2013)  Site-specific environmental regulations: e.g., Aituaki Environment Regulations 2006, Mitiaro Environment Regulations 2006 |
|  | Kiribati (2016) | X | [2015-2020](https://kiribati-data.sprep.org/resource/nissap-2014-2020) | Kiribati National Development Plan (2016-2019)  Kiribati Joint Implementation Plan for Climate Change and Disaster Risk Management  Kiribati National Fisheries Policy (2013-2023)  Kiribati Integrated Environment Policy (KIEP)  Kiribati Joint Implementation Plan  Agriculture Strategic Plan  Kiribati National Tourism Action Plan |
|  | Federated States of Micronesia (2018) | X | [2015-2020](https://kiribati-data.sprep.org/dataset/national-invasive-species-strategy-and-action-plan-2015-2020) | A Blueprint for Conserving the Biodiversity of the Federated States of Micronesia  State-wide Biodiversity Strategy and Action Plan  Biosecurity Law (DRAFT) 2006 |
|  | Nauru (2016) | X |  | National Environment and Development Management Strategies  Nauru Rehabilitation Programme  National Biodiversity Policy (DRAFT)  National Sustainable Development Strategy |
|  | Niue (2015) | X | [2013-2020](https://niue-data.sprep.org/dataset/niues-national-invasive-species-strategy-and-action-plan-2013-2020) | Niue National Strategic Plan (2014-2019)  Eco-tourism Guidelines (2014)  National Environment Management Strategy  Convention for the Protection of the Natural Resources and Environment of the South Pacific Region (1986) |
|  | Palau (2018) | X | [2018 - 2022](https://palau-data.sprep.org/resource/palaus-national-invasive-species-and-biosecurity-strategic-action-plan-2018-2022) | Cartagena Protocol on Biosafety  National Sustainable Land Management Policy  National Protected Areas Network (PAN) Management Strategy and Action Plan |
|  | Papua New Guinea (2019-2024) | X |  | PNG 40-year Development Strategic Plan (2010 – 2050)  Vision 2050  PNG Development Strategic Plan (2010-2030)  PNG Medium-Term Development Plan (2018-2020)  Sustainable Development Goals  Sustainable Land Use Policy (2014)  Policy on Protected Areas (2014)  National Climate Change Compatible Development Management Policy (2014) |
|  | Samoa (2016) | X | [2019-2024](https://www.sprep.org/attachments/VirLib/Regional/nissap-samoa-2019-2024.pdf) | Samoa National Environmental Sector Plan  Strategy for the Development of Samoa  Gangwon Declaration on Biodiversity for Sustainable Development  S.A.M.O.A Pathway  The Maritime Zones Act (1999)  The Sustainable Management of Biodiversity Policy (2007)  National Deforestation Policy |
|  | Solomon Islands (2016-2020) | X | 2019 (Draft) | Fisheries Management Act (2015)  Biosecurity Act (2013)  Environment Act (1998)  Solomon Islands National Development Strategy (2011-2020)  Climate Change Policy  National Adaptation Plan of Action (NAPA)  Solomon Islands Ridge to Reef Strategic Action Framework |
|  | Marshall Islands (2017) | X | [2016-2021](https://www.sprep.org/attachments/VirLib/Marshall_Islands/national-invasive-species-strategy-action-plan-2016-2021.pdf) | Strategic Development Plan (SDP) Framework (2003-2018)  Reimaanlok – National Conservation Area Plan |
|  | Tonga | X | [2021-2027](https://brb.sprep.org/sites/default/files/2023-07/NISSAP-Tonga.pdf) | Tonga Strategic Development Framework II (2015 – 2025)  Tonga Forest operation Plan (2014-2020)  Tonga Agriculture Sector Plan (2016-2020)  Tonga Fisheries Sector Plan (2016-2024)  Management Plan for Forest and Tree Resources of Tonga 2017  Tonga National Invasive Species Strategic Action Plan 2014-2020  Tonga Energy Strategy  Tonga Joint National Action Plan 2 for Climate Change and Disaster Risk Management |
|  | Tuvalu (2014) | X |  | Environment Protection Act 2008  National Ballast Water Management Strategy 2016-2020  Biosecurity Act 2017 (Act 24, 2017)  National Environmental Management Strategy (1997) |
|  | Vanuatu (2018) | X | [2014-2020](https://www.sprep.org/attachments/VirLib/Vanuatu/nissap-2014-2020.pdf) | Vanuatu National Sustainable Development Plan (2016-2030)  Vanuatu National Environment Policy and Implementation Plan (2016-2030)  National Sustainable Development Goals and Policies (2016-2030): Environment Pillar  National Land Use Planning Policy  Fisheries Act  Forestry Act  Sustainable Tourism Policy |
| **Plan** | **Country** | | **Year** | **Linked Domestic Frameworks/Strategies/Policies to Plan** |
| Invasive Species Strategy and Action Plan | American Samoa (US Territory) | | [2017](https://library.sprep.org/sites/default/files/asispp-plan-as.pdf) | Forest Action Plan  Marine Conservation Plan (2021-2024)  State and Wildlife Action Plan  Climate Change Framework |
| Biodiversity Conservation Plan | New Caledonia | | 2018 | 2030 National Strategy for Protected Areas |

**Table S3.B. Invasive Species Related International and Regional Frameworks/Policies**

| **Frameworks** | **Publish Year** | **International/Regional Publishing Institution** | **Coverage** |
| --- | --- | --- | --- |
| The guiding framework for invasive species management in the Pacific - A framework for managing invasive species and biosecurity in the Pacific Islands (Second Edition) | 2023 | SPREP | Regional |
| [Framework for Nature Conservation and Protected Areas in the Pacific Islands Region (2021 – 2025)](https://library.sprep.org/sites/default/files/2021-11/2021-2025-Framework-nature-conservation-EN.pdf) | 2021 | SPREP | Regional |
| [Pacific Islands Framework for Action on Climate Change](https://www.sprep.org/attachments/Publications/PIFACC-ref.pdf) (PIFACC) (2006 – 2015) | 2006 | SPREP | Regional |
| Aichi Biodiversity Targets (2011 - 2020) | 2011 | Convention on Biological Diversity (CBD) | Global |
| Sustainable Development Goals (SDGs) (2015-2030) | 2015 | United Nations (UN) | Global |
| CBD Strategic Plan for Biodiversity (2011 - 2020) | 2011 | Convention on Biological Diversity (CBD) | Global |
| Nagoya Protocol on Access to Genetic Resources and the Fair and Equitable Sharing of Benefits Arising from their utilization (Nagoya Protocol) | 2010 | Convention on Biological Diversity (CBD) | Global |
| Kunming-Montreal Global Biodiversity Framework | 2022 | Convention on Biological Diversity (CBD) | Global |
| United Nations Framework Convention on Climate Change (UNFCCC) | 1994 | United Nations (UN) | Global |
| United Nations Convention on the Law of the Sea (UNCLOS) | 1994 | United Nations (UN) | Global |
| International Plant Protection Convention (IPPC) Treaty | 1997 | Food and Agriculture Organization of the United Nations (FAO) | Global |
| Agreement on the Application of Sanitary and Phytosanitary Measures (SPS Agreement) | 1995 | World Trade Organization (WTO) | Global |
| International Trade in Endangered Species of Wild Fauna and Flora (CITIES) | 1973 | Convention on International Trade in Endangered Species of Wild Fauna and Flora (CITIES) | Global |
| International Ballast Water Management Convention | 2017 | International Maritime Organization (IMO) | Global |
| The Convention on Wetlands | 1975 | Ramsar | Global |

**APPENDIX S4: DEFINITIONS OF CLASSIFICATION ALGORITHMS AND SPECTRAL INDICES UTILISED TO DETECT AND MAP INVASIVE PLANT SPECIES**

| Classification Algorithm | Definition | | Source |
| --- | --- | --- | --- |
| Random Forest (RF) | A simple and flexible ensemble of tree predictors. RF can handle high-dimensional, noisy, and multi-source datasets with minimal overfitting, achieving high classification accuracy. | | (Breiman, 2001; Liu, Gong, Hu, & Gong, 2018) |
| Logistic Regression | LR models the a posteriori probability via a linear function of elements in the input feature space X while ensuring probabilities sum to one and remain in [0,1]. Often used for feature selection for binary classification in high-dimensional datasets. | | (Cheng, Varshney, & Arora, 2006) |
| Support Vector Machine (SVM) | Finds a hyperplane in N-dimensional space that classifies the data point distinctly. Hyperplanes are decision boundaries that help classify data points. Effective in high dimensional spaces and is versatile. | | (Gandhi, 2018) |
| Classification and Regression Trees (CART) | Identifies and constructs a binary decision tree using a sample of training data that is correctly classified. | | (Breiman, 2001) |
| Object-Based Image Analysis (OBIA) | Image segmentation based on the raster stack information. These segments are the basic units for classification and consist of homogenous regions. | | (Marzialetti et al., 2021) |
| Gradient Boosting Machines (GBM) | Based on the notion of boosting and is used to add new models to an ensemble. GBM constructs classification models which are introduced to an ensemble at each iteration. | | (Peerbhay, Germishuizen, Lottering, & Naicker, 2022) |
| Stochastic Gradient Boosting (SGB) | An efficient, modified GBM, where small classification trees are developed at each iteration. The trees are stacked together as a weighted sum of terms and each observation is classified according to the most common classification among the trees. | | (Peerbhay et al., 2022) |
| K-Nearest Neighbors (KNN) | A supervised machine learning algorithm that assumes similar data points are close to each other. | | (Harrison, 2018; Wu et al., 2019) |
| Neural Network (NN) | Elements in one set of data are associated with elements in a second set. NN transforms data from feature space to class space. | | (Atkinson & Tatnall, 1997) |
| Convolutional Neural Networks (CNN) | A deep learning technique that has been known to be accurate in image recognition. CNN automatically learns relevant patterns by firing multiple neurons to perceive spatial texture and context in an analogous manner, requiring minimal preprocessing to capture spatial properties. | | (Kattenborn et al., 2020) |
| *Linear Modelling Algorithms* | | | |
| Generalized Additive Model (GAM) | An extension of GLM that can handle non-linear relationships between response and multiple exploratory variables using nonparametric smoothing functions. | | (Huang, Peng, Huang, Deng, & Liu, 2022) |
| Hue, Intensity, Saturation (HIS) | Transformation algorithms developed for converting RBG values into parameters of human color perception | | (Duncan, Podest, Esler, Geerts, & Lyons, 2023) |
| *Spectral Vegetation Indices* | | | |
| Normalized Difference Vegetation Index (NDVI)  $\frac{\left( \boldsymbol{NIR- RED} \right)}{\boldsymbol{(NIR+ RED)}}$ | A measure of vegetation health and involves the use of the red (which vegetation has a low reflectance towards) and near infrared (which vegetation has a high reflectance towards) band in a normalized difference equation. | | (Saba, Latifi, Valadan Zoej, & Esmaili, 2022) |
| Enhanced Vegetation Index (EVI)  $\mathbf{2.5 x}\frac{\mathbf{(NIR-RED)}}{\boldsymbol{NIR+(6*RED-7.5*BLUE)+1}}$ | Quantifies vegetation health, however EVI corrects for environmental factors such as atmospheric conditions, soil, and canopy background noise. The index is also sensitive in areas with dense vegetation. A constant term, the soil adjustment factor L (equal to 1). | | (Matsushita, Yang, Chen, Onda, & Qiu, 2007) |
| Normalized Difference Wetness Index (NDWI)  $\frac{\left( \boldsymbol{GREEN-NIR} \right)}{\boldsymbol{(GREEN+ NIR)}}$ | A measure of plant water content plant and is particularly sensitive in grassland vegetation and responds quickly to growing grasses. | | Abdullah, Skidmore, Darvishzadeh, and Heurich (2019); Dahal et al. (2022); Peerbhay et al. (2022) |
| Normalized Difference Moisture Index (NDMI)  $\frac{\left( \boldsymbol{NIR-SWIR} \right)}{\boldsymbol{(NIR+SWIR)}}$ | A measure highly correlated with canopy water content and closely tracked changes in plant biomass and water stress, detecting lighter disturbances more significantly when compared with NDVI. | | (Jin & Sader, 2005) |
| Normalized Flower Red-Edge (NFRE)  $\frac{\left( \boldsymbol{RED EDGE-RED} \right)}{\boldsymbol{(RED EDGE+RED)}}$ | A red-edge index adapted to detect flowering of vegetation. | | Duncan et al. (2023); (McIntyre, 2015) |
| Normalized Difference Red-Edge (NDRE)  $\frac{\left( \boldsymbol{NIR- RED EDGE} \right)}{\boldsymbol{(NIR+ RED EDGE)}}$ | Analyzes the health of vegetation, particularly the amount of chlorophyll in plants. NDRE is most effective in the mid-to-late growing season. | | (Saba et al., 2022) |
| Green Normalized Difference Vegetation Index (GNDVI)  $\frac{\left( \boldsymbol{NIR- GREEN} \right)}{\boldsymbol{(NIR+ GREEN)}}$ | A vegetation index for estimating photosynthetic activity, commonly used to determine water and nitrogen uptake into plant canopy. | | (Hunt et al., 2010) |
| Soil Adjusted Vegetation Index (SAVI)  $\frac{\mathbf{NIR-RED}}{\mathbf{NIR+RED+L}}\boldsymbol{*(1+L)}$ |  | A transformation technique presented to minimize the influence of soil brightness in areas where negative cover is low in red and NIR bands. Describes dynamic soil-vegetation systems. | (Huete, 1988) |
| Tassel Cap Transformation:  Greenness Index  Brightness Index  Wetness Index (VIS+NIR+SWIR) |  | Enhances spectral information content by optimizing data for vegetation studies. The transformation consists of 6 multispectral features, the first three being brightness, greenness, and wetness, all of which can be differentiated across a multitemporal dataset. | (Jin & Sader, 2005) |
| *Texture-based statistical algorithms* | | | |
| Partial least squares-discriminant analysis (PLS-DA) | A set of binary variables describe the categorical variable on a set of predictor variables. Explanatory variables are decomposed into components that retain majority of necessary information to predict the dependent class variable of new samples. The aim of the PLA algorithm is dimension reduction. | | (Chetty, Mutanga, & Lottering, 2021) |
| Sparse partial least squares discriminant analysis (SPLS-DA) | Applies a scarcity solution that performs variable selection and dimensionality reduction in parallel. The approach is centered on PLS. | | (Lottering, Govender, Peerbhay, & Lottering, 2020) |
| Grey level co-occurring matrix (GLCM) | Describes the frequency of occurrence of individual pairs of values within an image. Then using this spatial relationship between neighboring pixels, certain features are used to describe certain texture. These filters or parameters are referred to as Haralick features. | | (Chetty et al., 2021) |
| Haralick Texture Features | These are the set of GLCM indicators: contrast, correlation, entropy, mean, dissimilarity, homogeneity, second moment and variance | | (Kupidura, 2019) |
| Laplace Filters | Derivative filters that identify areas of rapid change in imagery. The filter can be expressed as a convolution and are used to detect edges of objects in an image and parts of an image with high texture. | | (Kupidura, 2019) |
| *Dimensionality Reduction* |  |  |  |
| Principal Component Analysis (PCA) | Dimensionality reduction method used to reduce large data volumes by transforming these large variables into smaller, retraining most of the information | | (Abdi & Williams, 2010) |
| *Accuracy Assessment* |  | |  |
| Root Mean Square Error (RMSE)  $\sqrt{\frac{\boldsymbol{1}}{\boldsymbol{n}}\sum_{\boldsymbol{i=1}}^{\boldsymbol{n}} \frac{\boldsymbol{(}\mathbf{ŷ}\mathcal{i-}\mathbf{y}\mathcal{i}\boldsymbol{)}^{\boldsymbol{2}}}{\mathcal{n}}}$ | The square root of the mean of the square of all the error. A common general purpose error metric for numerical predictions, evaluating trained models for accuracy. RMSE estimates the standard deviation of the distribution of errors. | | (Kattenborn et al., 2020; Moody, 2019) |
